# Omega-3 Polyunsaturated Fatty Acid Intervention Against Established Autoimmunity in a Murine Model of Toxicant-Triggered Lupus

**DOI:** 10.3389/fimmu.2021.653464

**Published:** 2021-04-07

**Authors:** James J. Pestka, Peyman Akbari, Kathryn A. Wierenga, Melissa A. Bates, Kristen. N. Gilley, James G. Wagner, Ryan P. Lewandowski, Lichchavi D. Rajasinghe, Preeti S. Chauhan, Adam L. Lock, Quan-Zhen Li, Jack R. Harkema

**Affiliations:** ^1^Department of Microbiology and Molecular Genetics, Michigan State University, East Lansing, MI, United States; ^2^Department of Food Science and Human Nutrition, East Lansing, MI, United States; ^3^Institute for Integrative Toxicology, Michigan State University, East Lansing, MI, United States; ^4^Department of Pathobiology and Diagnostic Investigation, Michigan State University, East Lansing, MI, United States; ^5^Department of Biochemistry and Molecular Biology, Michigan State University, East Lansing, MI, United States; ^6^Department of Animal Science, Michigan State University, East Lansing, MI, United States; ^7^Department of Immunology and Internal Medicine, University of Texas Southwestern Medical Center, Dallas, TX, United States

**Keywords:** systemic lupus erythematosus, NZBWF1, ω-3 polyunsaturated fatty acid, silica, docosahexaenoic acid, ectopic lymphoid structure, glomerulonephritis, autoantibody

## Abstract

Workplace exposure to respirable crystalline silica dust (cSiO_2_) has been etiologically linked to the development of lupus and other human autoimmune diseases. Lupus triggering can be recapitulated in female NZBWF1 mice by four weekly intranasal instillations with 1 mg cSiO_2._ This elicits inflammatory/autoimmune gene expression and ectopic lymphoid structure (ELS) development in the lung within 1 week, ultimately driving early onset of systemic autoimmunity and glomerulonephritis. Intriguingly, dietary supplementation with docosahexaenoic acid (DHA), an ω-3 polyunsaturated fatty acid (PUFA) found in fish oil, beginning 2 week prior to cSiO_2_ challenge, prevented inflammation and autoimmune flaring in this novel model. However, it is not yet known how ω-3 PUFA intervention influences established autoimmunity in this murine model of toxicant-triggered lupus. Here we tested the hypothesis that DHA intervention after cSiO_2_-initiated intrapulmonary autoimmunity will suppress lupus progression in the NZBWF1 mouse. Six-week old NZWBF1 female mice were fed purified isocaloric diet for 2 weeks and then intranasally instilled with 1 mg cSiO_2_ or saline vehicle weekly for 4 consecutive weeks. One week after the final instillation, which marks onset of ELS formation, mice were fed diets supplemented with 0, 4, or 10 g/kg DHA. One cohort of mice (*n* = 8/group) was terminated 13 weeks after the last cSiO_2_ instillation and assessed for autoimmune hallmarks. A second cohort of mice (*n* = 8/group) remained on experimental diets and was monitored for proteinuria and moribund criteria to ascertain progression of glomerulonephritis and survival, respectively. DHA consumption dose-dependently increased ω-3 PUFA content in the plasma, lung, and kidney at the expense of the ω-6 PUFA arachidonic acid. Dietary intervention with high but not low DHA after cSiO_2_ treatment suppressed or delayed: (i) recruitment of T cells and B cells to the lung, (ii) development of pulmonary ELS, (iii) elevation of a wide spectrum of plasma autoantibodies associated with lupus and other autoimmune diseases, (iv) initiation and progression of glomerulonephritis, and (v) onset of the moribund state. Taken together, these preclinical findings suggest that DHA supplementation at a human caloric equivalent of 5 g/d was an effective therapeutic regimen for slowing progression of established autoimmunity triggered by the environmental toxicant cSiO_2_.

## Introduction

Systemic lupus erythematosus (lupus) is a chronic and debilitating autoantibody (AAb)-mediated systemic autoimmune disease affecting numerous organs including the kidney, lung, heart, skin, and brain (1). Patients with lupus characteristically have recurring cycles of flaring and remission that, over the long term, can result in irreversible organ damage. Environmental agents can contribute to onset, flaring, and progression of autoimmunity in genetically predisposed individuals (2). A prototypical example of an environmental autoimmune trigger is respirable crystalline silica dust (cSiO_2_), which is commonly inhaled by workers in mining, construction, and ceramics industries worldwide (3). Occupational exposure to cSiO_2_ has been etiologically associated with increased risk for the development and recurrent manifestation of lupus and other human autoimmune diseases (4–6), raising the need for preventive or therapeutic interventions for exposed workers.

cSiO_2_ triggered autoimmune flaring can be recapitulated by introducing the particle to lungs of mouse models of human lupus (7–13). Specifically relevant to the present investigation is the widely used lupus-prone female New Zealand Black White (F1) (NZBWF1) mouse, which typically displays autoantibody production early in life, glomerulonephritis and proteinuria at approximately age 34 weeks, and succumbs to the disease by age 52 week (14). When this model is subjected to 4 weekly intranasal instillations with 1 mg cSiO_2_, latency to onset for glomerulonephritis is reduced by 12 week (12, 13). Prior to glomerulonephritis onset, instilled cSiO_2_ evokes in these mice severe pulmonary pathology (i.e., alveolitis) that includes persistent alveolar accumulation of particle-laden macrophages, monocytes, necrotic phagocytic cells, nuclear and cytoplasmic debris, and neutrophilic inflammation. Additionally, there is accumulation of large numbers of T- and B-lymphoid cells, as well as IgG-secreting plasma cells, indicative of pathological ectopic lymphoid structures (ELS). Consistent with persistent particle-induced pulmonary inflammation and ELS neogenesis, lung lavage fluid and blood plasma from cSiO_2_-exposed mice exhibit elevated concentrations of proinflammatory cytokines, chemokines, and autoantibodies. Collectively, these findings support the contention that the lung functions as a nexus for cSiO_2_-triggered systemic autoimmune flaring and glomerulonephritis in NZBWF1 mice.

Lifestyle factors such as diet can also attenuate or potentiate autoimmunity. One potentially promising dietary intervention against lupus is the increased intake of the marine ω-3 polyunsaturated fatty acids (PUFAs) docosahexaenoic acid (C22:6 ω-3; DHA) and eicosapentaenoic acid (C20:5 ω-3; EPA) (15). Specifically, consumption of ω-3 PUFAs may ameliorate chronic inflammation and autoimmunity by (i) altering membrane function, (ii) modulating gene expression, (iii) competition with inflammatory ω-6 PUFA-generated eicosanoids, and iv) functioning as substrates for metabolism to pro-resolving mediators [reviewed in (16–18)]. Both preclinical (19–21) and clinical studies (18, 22–24) suggest that ω-3 PUFAs might be used to prevent onset of or counter ongoing lupus symptoms, including nephritis.

The specific use of ω-3 PUFAs as a preventive approach against cSiO_2_-triggered autoimmune flaring was demonstrated in a recent study in which female NZBWF1 mice consumed purified isocaloric diets supplemented with DHA at 0, 4, and 10 g/kg (13). These concentrations reflect on a caloric basis, human equivalent doses (HEDs) of 0, 2, and 5 g/d, respectively. DHA supplementation dramatically suppressed cSiO_2_-triggered B- and T-cell, follicular dendritic cell (FDC), and IgG^+^ plasma cell appearance in the lungs over the course of the experiment. Notably, pulmonary transcriptomic signatures of cSiO_2_-treated mice fed control diet reflected progressive amplification of inflammation-, chemokine-, and interferon (IFN)-related gene pathways, all of which were dose-dependently inhibited by DHA (25). Protein microarray profiling has further revealed that DHA feeding dose-dependently inhibited cSiO_2_-induced AAb responses against a multitude of lupus-associated autoantigens (AAgs), including histones, DNA, Smith antigen, complement, ribonucleoprotein, Ro/SSA, and La/SSB (26). Importantly, cSiO_2_-triggered glomerulonephritis and lymphoid cell accumulation in the renal cortex at 13 weeks post-instillation (PI) was prevented by DHA consumption (13).

Collectively, the above-described studies establish that DHA has potent prophylactic effects against cSiO_2_-triggered lupus in NZBWF1 mice. From a translational perspective, it is now imperative to understand how therapeutic ω-3 PUFA intervention after autoimmunity induction by cSiO_2_ influences subsequent disease progression in this lupus model. To address this question, we tested here the hypothesis that DHA intervention after cSiO_2_-initiated intrapulmonary autoimmunity will suppress lupus progression in the NZBWF1 mouse. Our results demonstrated that supplementation with DHA at 5 g/d but not 2 g/d DHA, after established pulmonary autoimmunity, was therapeutically efficacious in delaying cSiO_2_-induced systemic autoimmunity, glomerulonephritis, and mortality.

## Materials and Methods

### Mice

This study was approved by the Institutional Animal Care and Use Committee at Michigan State University (AUF #01/15-021-00). Female lupus-prone NZBWF1 mice at age 6-week were obtained from Jackson Laboratories (Bar Harbor, ME), assigned randomly to experimental groups, housed four per cage, and given free access to food and water throughout the experiment. Animal facilities were maintained at constant temperature (21–24°C) and constant humidity (40–55%) with a 12 h light/dark cycle.

### Diets

Three diet formulations were employed that were based on the purified American Institute of Nutrition (AIN)-93G diet containing 70 g/kg fat that was designed for optimal rodent nutrition (27) (Table 1). Each diet contained 10 g/kg corn oil to ensure adequate basal essential fatty acids. The control diet (CON) contained 60 g/kg high-oleic safflower oil (Hain Pure Food, Boulder, CO). For DHA diets, high-oleic safflower oil was substituted with 10 g/kg (low DHA) or 25 g/kg (high DHA) microalgal oil containing 40% DHA (DHASCO; provided by Dr. Kevin Hadley, DSM Nutritional Products, Columbia MD). Resultant experimental diets contained 0.4 and 1.0% DHA which reflect HEDs of 2 and 5 g per day, respectively. Final fatty acid concentrations of experimental diets are shown in Table 2. Experimental diets were prepared weekly and stored at −20°C until use to preclude lipid oxidation. Fresh diet was supplied *ad libitum* to mice every two days.

**Table 1:** Formulations of experimental diets^a^.

	**Experimental Diet**		
	**CON**	**low DHA^e^**	**high DHA^f^**
**Macronutrient**	**(g/kg total diet)**		
**Carbohydrates**			
Corn Starch	398	398	398
Maltodextrin (Dyetrose)	132	132	132
Sucrose	100	100	100
Cellulose	50	50	50
kcal (% of total)	63.2	63.2	63.2
**Proteins**			
Casein	200	200	200
L-Cysteine	3	3	3
kcal (% of total)	19.7	19.7	19.7
**Fats^a^**			
Corn Oil^b^	10	10	10
High-Oleic Safflower Oil^c^	60	50	35
DHA-enriched Algal Oil^d^	0	10	25
kcal (% of total)	17.1	17.1	17.1
**Other**			
AIN93G Mineral Mix	35	35	35
AIN93G Vitamin Mix	10	10	10
Choline Bitartrate	3	3	3
TBHQ Antioxidant	0.01	0.01	0.01

*Values shown are the mass amounts (g) per kg diet*.

a *Based on oil composition reported by the manufacturer*;

b *Corn oil contained 612 g/kg linoleic acid and 26 g/kg oleic acid*;

c *high-oleic safflower oil contained 750 g/kg oleic acid and 140 g/kg linoleic acid*;

d *Algal oil contained 395 DHA g/kg and 215 g oleic acid /kg*;

e *4g/kg diet, calorically equivalent to human DHA consumption of 2 g/d*;

f *10 g/kg diet, calorically equivalent to human DHA consumption of 5 g/d*.

**Table 2:** Fatty acid content of experimental diets.

		**Experimental Diet**		
		**CON**	**low DHA**	**high DHA**
**Common Name**	**Formula**	**(% total fatty acids)**		
Myristic	C14:0	0.31, 0.00	1.47, 0.04	3.38, 0.56
Palmitic	C16:0	4.46, 0.00	5.13, 0.04	5.86, 0.30
Palmitoleic	C16:1ω7	0.07, 0.00	0.31, 0.01	0.70, 0.11
Stearic	C18:0	1.59, 0.00	1.44, 0.00	1.27, 0.03
Elaidic	C18:1t	0.172, 0.00	0.11, 0.00	0.11, 0.01
Oleic	C18:1ω9	49.05, 0.06	44.06, 0.20	36.81, 2.42
Linoelaidic	C18:2ω6t	0.09, 0.00	0.08, 0.01	0.08, 0.00
Linoleic	C18:2ω6	12.96, 0.06	12.17, 0.01	10.08, 0.36
Arachidic	C20:0	0.27, 0.00	0.24, 0.00	0.20, 0.01
gamma-Linolenic	C18:3ω6	0.01, 0.00	0.01, 0.00	0.01, 0.00
Eicosenoic	C20:1ω9	019, 0.00	0.17, 0.00	0.15, 0.01
alpha-Linolenic	C18:3ω3	0.23, 0.01	0.21, 0.00	0.19, 0.00
Eicosadienoic	C20:2ω6	0.03, 0.00	0.03, 0.00	0.04, 0.01
Behenic	C22:0	0.19, 0.00	0.18, 0.00	0.17, 0.00
Arachidonic	C20:4ω6	0.00, 0.00	0.00, 0.00	0.01, 0.00
Lignoceric	C24:0	0.13, 0.00	0.12, 0.00	0.11, 0.00
Eicosapentaenoic	C20:5ω3	0.00, 0.00	0.01, 0.00	0.01, 0.00
Nervonic	C24:1ω9	0.11, 0.00	0.09, 0.00	0.07, 0.00
Docosapentaenoic ω3	C22:5ω3	0.00, 0.00	0.07, 0.00	0.17, 0.03
Docosahexaenoic	C22:6ω3	0.00, 0.00	4.07, 0.11	10.59, 1.82
∑ SFA		9.92, 0.01	8.41, 0.08	10.82, 0.82
∑ MUFA		70.77, 0.09	44.76, 0.19	37.85, 2.32
∑ω-6 PUFA		18.70, 0.08	12.30, 0.00	10.21, 0.35
∑ω-3 PUFA		0.34, 0.00	4.36, 0.11	10.96, 1.85
ω 6: ω 3 ratio		55.38, 0.38	2.82, 0.07	0.95, 0.19

*Data presented as percent of total fatty acids as measured by GLC. Data are mean ± SEM, n = 2*.

### cSiO_2_ Preparation

Min-U-Sil-5 (1.5-2.0 μm average particle size, U.S. Silica, Berkeley Springs, WV) was prepared as previously described (28) by suspending in 1M HCl for 1 h at 100°C. After cooling, cSiO_2_ was washed with sterile water three times and dried at 200°C overnight. Stock suspensions were prepared fresh in phosphate buffered saline (PBS) before exposure, sonicated, and vortexed for 1 min prior to intranasal instillation.

### Experimental Design

**Figure 1:**
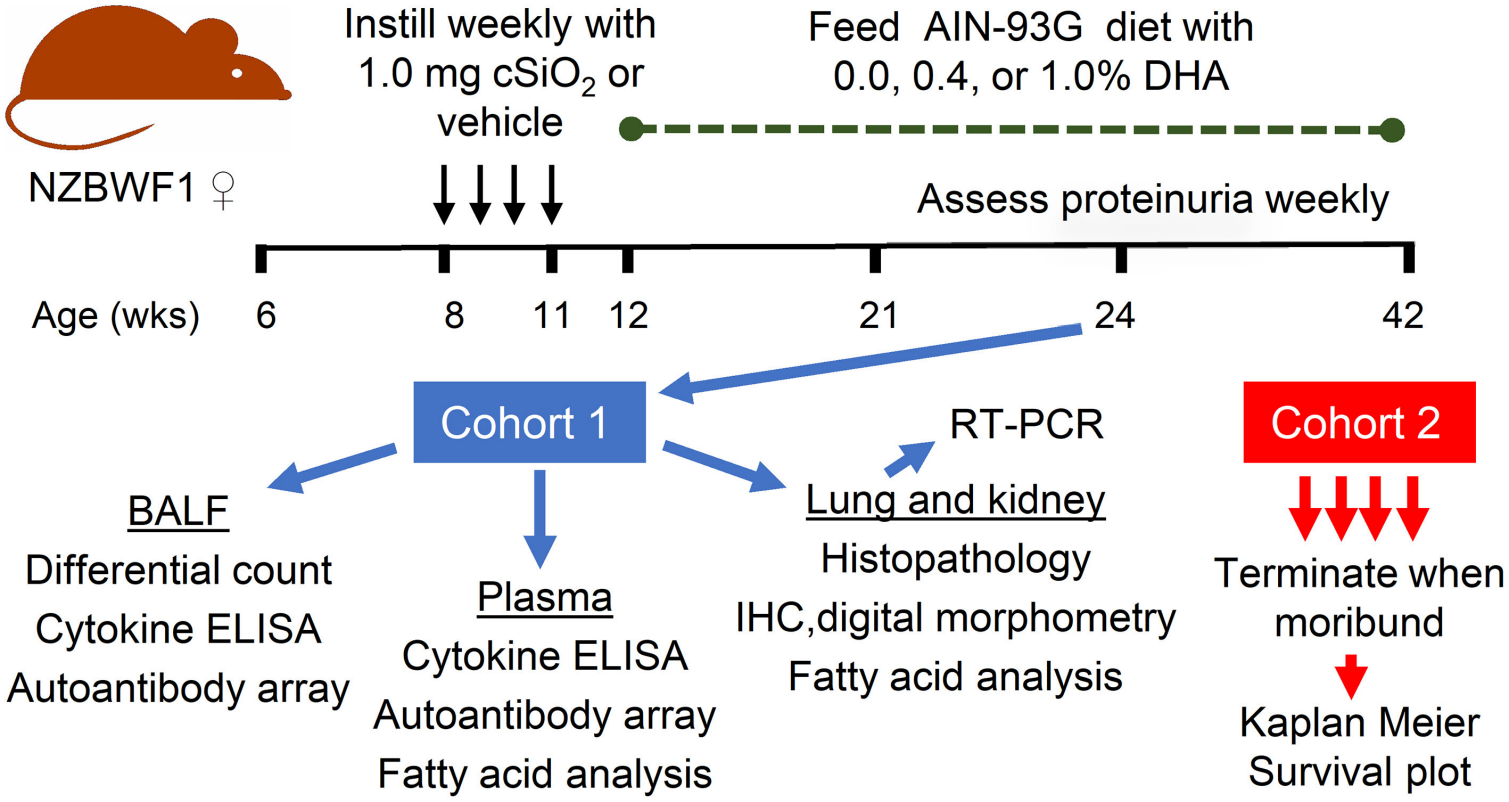
Experimental design. Female NZBWF1 were obtained at 6 week of age and initiated on AIN-93G diet containing 0.0% DHA. At age 8 week, mice were intranasally instilled with 1.0 mg cSiO_2_ or Veh once per week for 4 weeks. One week following the final cSiO_2_ instillation, animals were either maintained on the control diet or fed diets containing 0.4 or 1% DHA (w/w). Eight animals per exposure/diet group (cohort 1) were sacrificed at 24 weeks, at which time BALF was collected for differential cell counts, cytokine ELISAs, and assessment of autoantibodies; plasma collected for cytokine ELISAs, assessment of autoantibodies, and fatty acid analysis; lungs and kidneys collected for histopathology, IHC and digital morphometry, RT-PCR, and fatty acid analysis. The remaining 8 animals per exposure/diet group (cohort 2) were monitored until moribund, at which point they were humanely euthanized. During this time, proteinuria was assessed weekly as a measure of disease progression.

The overall experimental design of this study is depicted in Figure 1. Briefly, 64 6-week old female NZBWF1 mice were maintained on CON diet for 7 week. At age 8-week, mice were anesthetized with 4% isoflurane and intranasally instilled with 1.0 mg cSiO_2_ in 25 μl PBS (*n* = 48) or 25μl PBS vehicle (Veh) (*n* = 16) as described previously (12). Treatments were repeated weekly for 4 week s. This dosing regimen reflects approximately one half of the human lifetime permissible exposure level for cSiO_2_ at the recommended Occupational Safety and Health Administration limit (29). Experimental diets were initiated 1 week PI which, as reported previously (13), corresponds with the onset of pulmonary autoimmunity reflected by ELS development. Groups of mice were assigned to diets as follows: (i) Veh-treated mice fed CON diet (Veh/CON), *n* = 16), (ii) cSiO_2_-treated mice fed CON diet (cSiO_2_/CON, *n* = 16), (iii) cSiO_2_-treated mice fed low DHA diet (cSiO_2_/low DHA, *n* = 16), and (iv) cSiO_2_-treated mice fed high DHA diet (cSiO_2_/high DHA, *n* = 16). Mice were divided into two equivalent cohorts and maintained on assigned diets until the end of each respective experiment.

Experimental Cohort 1 mice (*n* = 8/group) were terminated 12 weeks after the last cSiO_2_ instillation. This termination time corresponds with robust autoimmune pathogenesis in the lung, systemic autoimmunity, and initiation of glomerulonephritis observed previously in NZBWF1 mice subjected to the above cSiO_2_ exposure regimen (12, 13, 30). Animals were anesthetized by intraperitoneal injection with 56 mg/kg body weight sodium pentobarbital and euthanized by exsanguination via the abdominal aorta. Blood was obtained with heparin-coated syringes and centrifuged at 3500 *x* g for 10 min at 4°C to obtain plasma, which was stored at−80°C for fatty acid analyses and AAb microarray. Bronchoalveolar lavage fluid (BALF) was collected from whole lungs and centrifuged at 2400 *x* g for 15 min as described previously (31). The cell fraction was subjected to inflammatory cell differential analysis and the supernatant fraction was stored at−80°C for cytokine ELISAs and autoantibody microarray. After lavage, the right lung lobes were removed, frozen in liquid nitrogen, and stored at−80°C for fatty acid analysis and FDC immunohistochemistry (IHC). Caudal lung lobes were removed, held in RNAlater (Thermo Fisher Scientific, Wilmington, DE) for 16 h at 4°C, and then stored at−80°C until RNA isolation. The left lung lobe was fixed with 10% neutral buffered formalin (Fisher Scientific, Pittsburgh, PA) at constant pressure (30 cm H_2_O) for minimum of 1 h and stored in fixative until further processing for histology and B- and T-cell IHC. The right kidney was excised, and the cranial portion fixed in 10% neutral buffered formalin for 24 h for histopathology and IHC. The left kidney was frozen at−80°C until fatty acid analysis.

Experimental Cohort 2 mice (*n* = 8/group) remained on experimental diets to monitor glomerulonephritis progression and survival. Mice were assessed weekly for proteinuria using dipsticks (Cortez Diagnostics, Inc., Calabasas, CA). They were further evaluated daily for established moribund criteria that included weight loss, inactivity, ruffled fur, and temperature drop (32). After reaching a moribund state, individual mice in this cohort were humanely euthanized as described for Cohort 1.

### Fatty Acid Analyses

Gas liquid chromatography (GLC) analysis of fatty acids in diet and plasma was performed at OmegaQuant Analytics, LLC (Sioux Falls, SD) which is an independent CLIA-certified laboratory. Fatty acid concentrations of lung and kidney were determined at Michigan State University using a modification of a direct transesterification method (33) as reported previously (34). Fatty acid data were presented as percent (w/w) of fatty acids detected with a chain length between 10 and 24 carbon atoms. The lower limit of detection was <0.001 g/100 g fatty acids. The ω-3 HUFA score and %EPA+DHA were determined for tissue samples. The ω-3 HUFA score is the sum of EPA (C20:5ω3), DPA (C22:5ω3), and DHA (C22:6ω3) as a percentage of the most abundant HUFAs (C20:5ω3, C22:5ω3, C22:6ω3, C20:3ω6, C20:4ω6, C22:4ω6, C22:5ω6, C20:3ω9) (35). The %EPA+DHA was calculated by taking the sum of EPA and DHA as a percent of total fatty acids (36). In red blood cells, this value is referred to as Omega 3 Index.

### BALF Cellularity

BALF cell pellets were resuspended in PBS and total leukocyte numbers were measured using a hemocytometer. Cytological slides were prepared by centrifugation at 40 *x* g for 10 min and stained with Diff-Quick (ThermoFisher). Differential cell counts of macrophages, neutrophils, eosinophils, and lymphocytes were determined from a minimum of 200 cells in cytospin slides.

### Cytokine Analyses

DuoSets (R&D Systems, Minneapolis, MN) were used to analyze BALF supernatants for TNF-α, MCP-1, and B-cell activating factor (BAFF) according to manufacturer's instructions.

### Histopathology of Lung and Kidney

Randomly oriented, serial sections of formalin-fixed left lung lobes were routinely processed and embedded in paraffin. Tissue sections (5 μm thickness) were deparaffinized and stained with hematoxylin and eosin (H&E) for light microscopic examination of histopathology. Severity of lung lesions were scored semi-quantitatively by a board certified veterinary pathologist without knowledge of individual animal exposure history (i.e., in a blinded manner). Scoring of histopathology included the amount of: (a) lymphoid aggregates within perivascular and peribronchiolar regions; (b) ELS; (c) alveolar proteinosis; (d) alveolitis (defined as the increased accumulation in the alveolar parenchyma of neutrophils, lymphocytes, and mononuclear/macrophages); (e) alveolar type II epithelial cell hyperplasia; and (f) mucous cell metaplasia in bronchiolar epithelium. Individual lungs were semi-quantitatively graded for these lesions as % of total pulmonary tissue examined based on the following criteria: (0) no changes compared to control mice; (1) minimal (<10%); (2) slight (10–25%); (3) moderate (26–50%); (4) severe (51–75%); or (5) very severe (>75%) of total area affected.

Fixed kidneys were similarly processed for light microscopic examination and tissue sections were stained with H&E or Periodic acid-Schiff and hematoxylin (PASH). Severity of lupus nephritis was evaluated in a blinded manner by the board-certified veterinary pathologist using a modified International Society of Nephrology/Renal Pathology Lupus Nephritis Classification system (37). Renal histopathology was semi-quantitatively graded as follows: (0) no tubular proteinosis and normal glomeruli; (1) mild tubular proteinosis with multifocal segmental proliferative glomerulonephritis and occasional early glomerular sclerosis and crescent formation; (2) moderate tubular proteinosis with diffuse segmental proliferative glomerulonephritis, early glomerular sclerosis and crescent formation; and (3) marked tubular proteinosis with diffuse global proliferative and sclerosing glomerulonephritis.

### Immunohistochemistry and Digital Morphometry of Lung and Kidney

Immunohistochemistry was performed as described previously by Bates et al. (12, 30). Briefly, formalin-fixed, paraffin embedded lung and kidney tissue sections were employed for identification of B cells using 1:600 rat anti-CD45R monoclonal antibody (Becton Dickinson, Franklin Lakes, NJ, catalog # 550286) and T cells using 1:250 rabbit anti-CD3 polyclonal antibody (Abcam, Cambridge, MA catalog # ab5690). FDCs were detected in frozen lung and kidney sections using 1:500 rat anti-CD21/35 monoclonal antibody (Becton Dickinson; catalog # 553817). Slides were digitally scanned using a VS110 (Olympus, Hicksville, NY) virtual slide system. At least 100 digital images were then captured at 20X magnification using systematic random sampling with NewCast software (Visiopharm, Hoersholm, Denmark). Volume densities of CD3^+^, CD45R^+^, or CD21/35^+^ cells were estimated using a point grid over the randomly sampled images with the STEPnizer 1.8 Stereology Tool. The number of points landing directly on the CD45R^+^, CD3^+^, or CD21/35^+^ cells were counted and the percent positive per reference area was calculated.

### mRNA Expression

TriReagent (Sigma Aldrich, St. Louis, MO) was used to extract total RNA from the lung. RNA was further purified with a Zymo RNA Clean and Concentrator Kit with DNase digestion (Zymo Research, Irvine, CA, catalog #R1017). Total RNA was measured with a NanoDrop-1000 (Thermo Fisher Scientific) and reverse transcribed to cDNA at 50 ng/μl using a High-Capacity cDNA Reverse Transcription Kit (Thermo Fisher Scientific, Waltham, MA). TaqMan Assays were carried out using a SmartChip Real-Time PCR System in technical triplicates for inflammation-associated genes (*Il1a, Il1b, IL6, Il18,Tnfa, Stat2, Pparg, Nos2,Nrlp3, Nfkb, Tlr4, Tlr7, Tlr8,Tlr9, Zbp1, Arg1*), chemokine-related genes (*Ccl2, Ccl7, Ccl8, Ccl12, Cxcl1, Cxcl2, Cxcl3, Cxcl5, Cxcl9, Cxcl10*), IFN-regulated genes (*Ifit1, Irf7, IsG15, Mx1, Ifi44, Oas1a, Oas2, Oasl1, Psmb8, Rsad2, Siglec1)*, and 3 housekeeping genes (*Gapdh, Hprt, Actb*). Gene expression levels were normalized to the endogenous housekeeping genes and reported as fold change relative to the experimental control group using the 2^−ΔΔCT^ method (38).

### High-Throughput Protein Microarray AAb Profiling

High-throughput profiling of IgG AAb in BALF and plasma against a broad range of autoantigens (AAgs) was performed at the Microarray and Immune Phenotyping Core Facility at The University of Texas Southwestern Medical Center using AAg coated protein arrays (26, 39). Briefly, plasma and BALF samples were pre-treated with DNAse I to remove free-DNA, then samples were diluted at 1:50 (for plasma) or 1:25 (for BALF) and hybridized to protein array plates coated with 122 antigens and 6 controls. The antibodies binding with the antigens on plate were detected with Cy3-conjugated anti-mouse IgG (1:2000, Jackson ImmunoResearch Laboratories, PA) and fluorescent images captured with a Genepix 4200A scanner (Molecular Devices, CA). Fluorescent images were transformed to signal intensity values using GenePix 7.0 software and background-subtracted and normalized to internal controls for IgG. The processed signal intensity value for each AAb was reported as antibody score (Ab-score), which is expressed based on the normalized signal intensity and signal-to-noise ratio (SNR) using the formula:

$$\begin{array}{l} \text{Ab}-\text{score}=log_{2}\left(NSI^{*}SNR+1\right). \end{array}$$

Normalized and unit variance-scaled Ab-score values were represented with heat maps, organized by unsupervised hierarchical co-clustering (HCC) generated using ClustVis (40). In heat maps, values were centered by rows; imputation was used for missing value estimation. Rows were clustered using Euclidean distance and Ward linkage. A volcano plot was generated in R Shiny online tool (https://paolo.shinyapps.io/ShinyVolcanoPlot/); *p*-values and fold change values for the volcano plots were calculated using Microsoft Excel 2016. Selected Ab-scores are reported as scatter plots, generated using GraphPad Prism version 8.3.0 for Windows (GraphPad Software, San Diego, California USA, www.graphpad.com).

### Data Analysis and Statistics

All statistical analyses were performed using GraphPad Prism version 8.3.0. Data were inspected using the Grubb's outlier test (with *Q* = 1%) to identify potential outliers and assessed for normality using the Shapiro-Wilk test (*p* < 0.01). Non-normal and semi-quantitative were analyzed using the Kruskal-Wallis nonparametric test with Dunn's *post-hoc* test (for multiple groups) or Mann-Whitney U test (for two groups). Normal data with unequal variance were analyzed using a Browns-Forsythe and Welch analysis of variance (ANOVA) with Dunnett's T3 *post-hoc* test or Welch's T-test. Data meeting both normality and equal variance assumptions were analyzed using a one-way ANOVA with Dunnett's *post-hoc* test or unpaired Student's *t*-test. Data were presented as mean ± standard error of the mean (SEM). A *p*-value ≤ 0.05 was considered statistically significant.

## Results

### Body Weight and Tissue Fatty Acid Content

**Figure 2:**
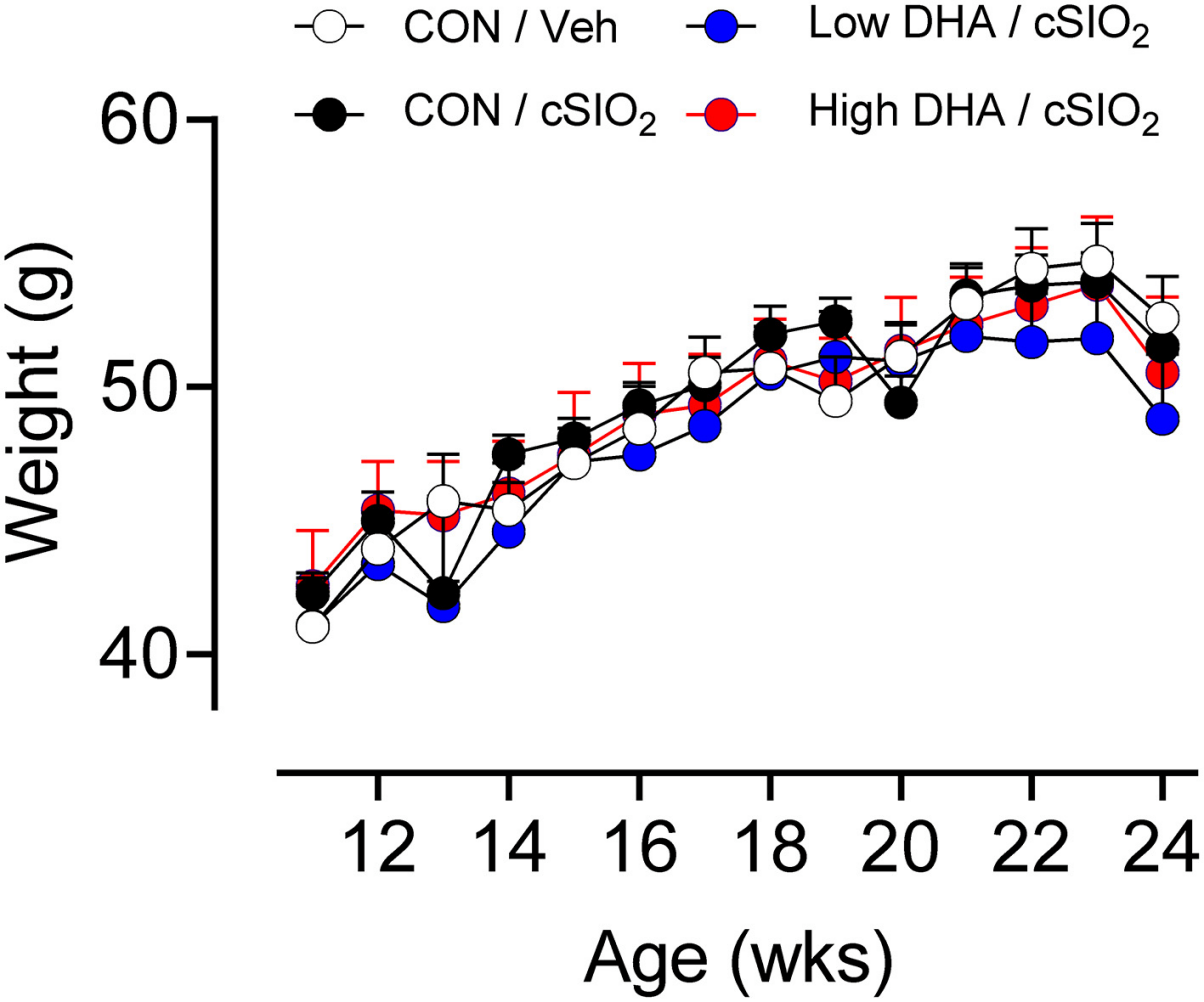
Body weight change in NZBWF1 mice is not affected by cSiO_2_ treatment and experimental diets. Animals in Cohort 1 were weighed weekly following the final cSiO_2_ instillation. Data presented as mean± SEM. Differences between CON/Veh and CON/cSiO_2_ were assessed by Student's *T*-test. Low DHA/cSiO_2_ and high DHA/cSiO_2_ were compared to CON/cSiO_2_ using one-way ANOVA followed by Dunnett's *post-hoc* test. At each time point, we observed no significant differences between treatment groups.

**Figure 3:**
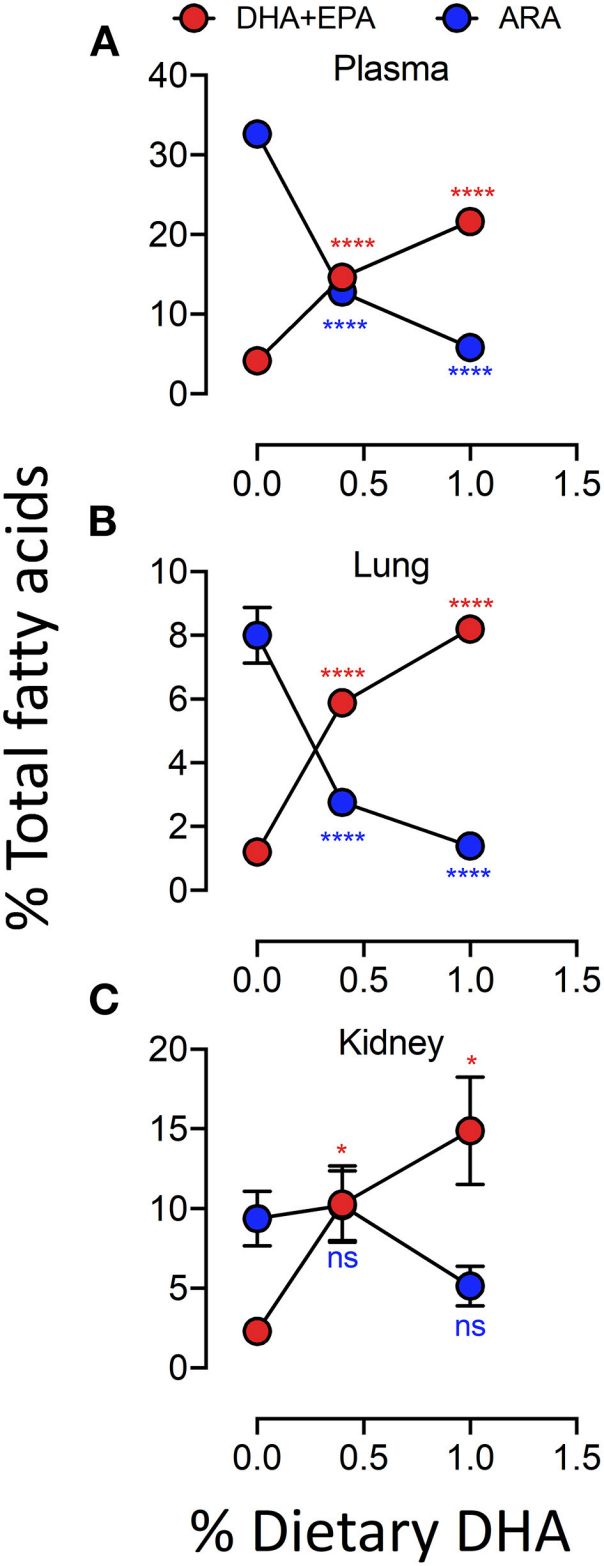
Experimental diets influence the DHA and ARA concentrations in kidney, lung and plasma. Incorporation of EPA and DHA in the plasma **(A)** and tissues **(B,C)** increased in a dose-dependent manner that coincided with a reduction in ARA. Data presented as mean ± SEM. Fatty acid levels from animals fed the low-and high-DHA diets were compared to animals fed the CON diet. Statistical comparisons were performed as described in the Methods section. *p < 0.05, ****p < 0.0001, ns = not significant. Asterisk/text color corresponds to fatty acid data being analyzed (ARA, red; EPA+DHA, blue).

Female NZBWF1 mice were treated with cSiO2 and fed experimental diets as indicated in Figure 1. Body weight increases in Cohort 1, which were terminated 13 week post final cSiO_2_ instillation (PI), were unaffected by cSiO_2_ treatment or diet composition (Figure 2). Replacing high oleic safflower oil in AIN-93G diets with increasing amounts of DHA dose-dependently increased DHA content of the plasma (Figure 3A; Table 3), lung (Figure 3B; Supplementary Table 1), and kidney (Figure 3C; Supplementary Table 2) in NZBWF1 mice 12 week after initiating experimental diets. cSiO_2_ exposure had negligible effects on tissue DHA content of CON-fed mice. Concurrently, there were nearly equivalent reductions of the ω-6 PUFA arachidonic acid (ARA), indicating that DHA replaced ARA in these tissues. Finally, EPA also increased dose-dependently in plasma (Table 3), lung (Supplementary Table 1), and kidney (Supplementary Table 2) with increasing concentrations of DHA in the diet. Recent isotope labeling studies have revealed increased EPA in DHA-fed rats is primarily a result of elongation/desaturation of alpha linoleic acid (ALA) to docosapentaenoic acid (DPA) mediated via feedback inhibition by DHA and, to a much lesser extent, by retroconversion of DHA (41).

**Table 3:** Plasma fatty acid content of experimental groups.

		**Experimental Group**			
		**Veh/CON**	**cSiO_2_/CON**	**cSiO_2_/low DHA**	**cSiO_2_/high DHA**
**Common Name**	**Formula**	**(% of total fatty acids)**			
Myristic	C14:0	0.05, 0.01	0.07, 0.01^#^	0.07, 0.02	0.11, 0.05
Palmitic	C16:0	15.25, 0.39	15.72, 0.69	17.00, 1.23	17.90, 1.53^**^
Palmitoleidic	C16:1ω7t	0.02, 0.00	0.02, 0.01	0.02, 0.01	0.01, 0.01^*^
Palmitoleic	C16:1ω7c	1.29, 0.19	1.42, 0.15	1.16, 0.20^*^	1.49, 0.17
Stearic	C18:0	12.57, 0.51	12.04, 0.41^#^	11.99, 1.32	11.02, 1.04
Elaidic	C18:1ω9t	0.10, 0.01	0.10, 0.02	0.09, 0.02	0.07, 0.01^**^
Oleic	C18:1ω9c	14.39, 0.91	17.43, 3.17^#^	16.37, 2.91	16.06, 1.52
Linoelaidic	C18:2ω6t	0.14, 0.01	0.15, 0.02	0.12, 0.02	0.10, 0.03^**^
Linoleic	C18:2ω6c	10.41, 1.53	12.32, 1.66^#^	21.72, 1.31^***^	22.16, 1.71^***^
Arachidic	C20:0	0.03, 0.01	0.04, 0.01	0.05, 0.01^*^	0.04, 0.01
Gamma-linolenic	C18:3ω6	0.21, 0.03	0.23, 0.04	0.16, 0.04^***^	0.13, 0.03^***^
Eicosanoic	C20:1ω9	0.30, 0.04	0.32, 0.03	0.33, 0.05	0.28, 0.08
Linolenic	C18:3ω3	0.01, 0.00	0.02, 0.01	0.02, 0.01	0.02, 0.01
Eicosadienoic	C20:2ω6	0.12, 0.03	0.12, 0.01	0.24, 0.07^**^	0.17, 0.07
Benehinic	C22:0	0.39, 0.09	0.35, 0.09	0.27, 0.14	0.19, 0.10^*^
Dihomo-Gamma-linolenic	C20:3ω6	1.29, 0.14	1.16, 0.14	2.42, 0.59^**^	2.03, 0.66^*^
Arachidonic	C20:4ω6	37.14, 1.96	32.63, 4.35^#^	12.80, 2.44^***^	5.84, 1.31^***^
Lignoceric	C24:0	0.02, 0.01	0.02, 0.00	0.02, 0.01	0.02, 0.01
Eicosapentaenoic	C20:5ω3	0.03, 0.00	0.04, 0.01^#^	2.75, 1.08^**^	6.70, 2.41^***^
Nervonic	C24:1ω9	0.20, 0.05	0.20, 0.05	0.21, 0.08	0.20, 0.08
Adrenic	C22:4ω6	0.23, 0.02	0.26, 0.04	0.05, 0.01^***^	0.02, 0.01^***^
Docosapentaenoic ω6	C22:5ω6	1.28, 0.12	1.15, 0.26	0.02, 0.01^*^	0.01, 0.01^***^
Docosapentaenoic ω3	C22:5ω3	0.06, 0.01	0.07, 0.03	0.26, 0.04^***^	0.42, 0.06^***^
Docosahexaenoic	C22:6ω3	4.44, 0.14	4.12, 0.24^##^	11.87, 0.54^***^	14.95, 1.65^***^
∑ SFA		28.33, 0.24	28.24, 0.56	29.40, 0.61^**^	29.28, 0.75^**^
∑ MUFA		16.31, 1.12	19.49, 3.15^#^	18.17, 2.78	18.11, 1.49
∑ n-3 PUFA		4.54, 0.13	4.25, 0.21^##^	14.91, 0.96^***^	22.10, 1.40^***^
∑ n-6 PUFA		50.82, 0.90	48.02, 3.07^#^	37.52, 2.29^***^	30.51, 2.28^***^
EPA+DHA		4.47, 0.14	4.16, 0.24^##^	14.62, 0.97^***^	21.65, 1.36^***^
% ω3 in HUFA		10.18, 0.44	10.83, 1.21	49.29, 4.89^***^	73.39, 4.71^***^

*Determined by GLC. All fatty acids represented as percent of total fatty acids and as mean ± SEM, n = 8. Number signs indicate significantly different than Veh/CON*:

# *p < 0.05*,

## *p < 0.01. Asterisks indicate significantly different than cSiO_2_/CON*:

* *p < 0.05*,

** *p < 0.01*,

*** *p < 0.001*.

### BALF Cellularity and Cytokines

**Figure 4:**
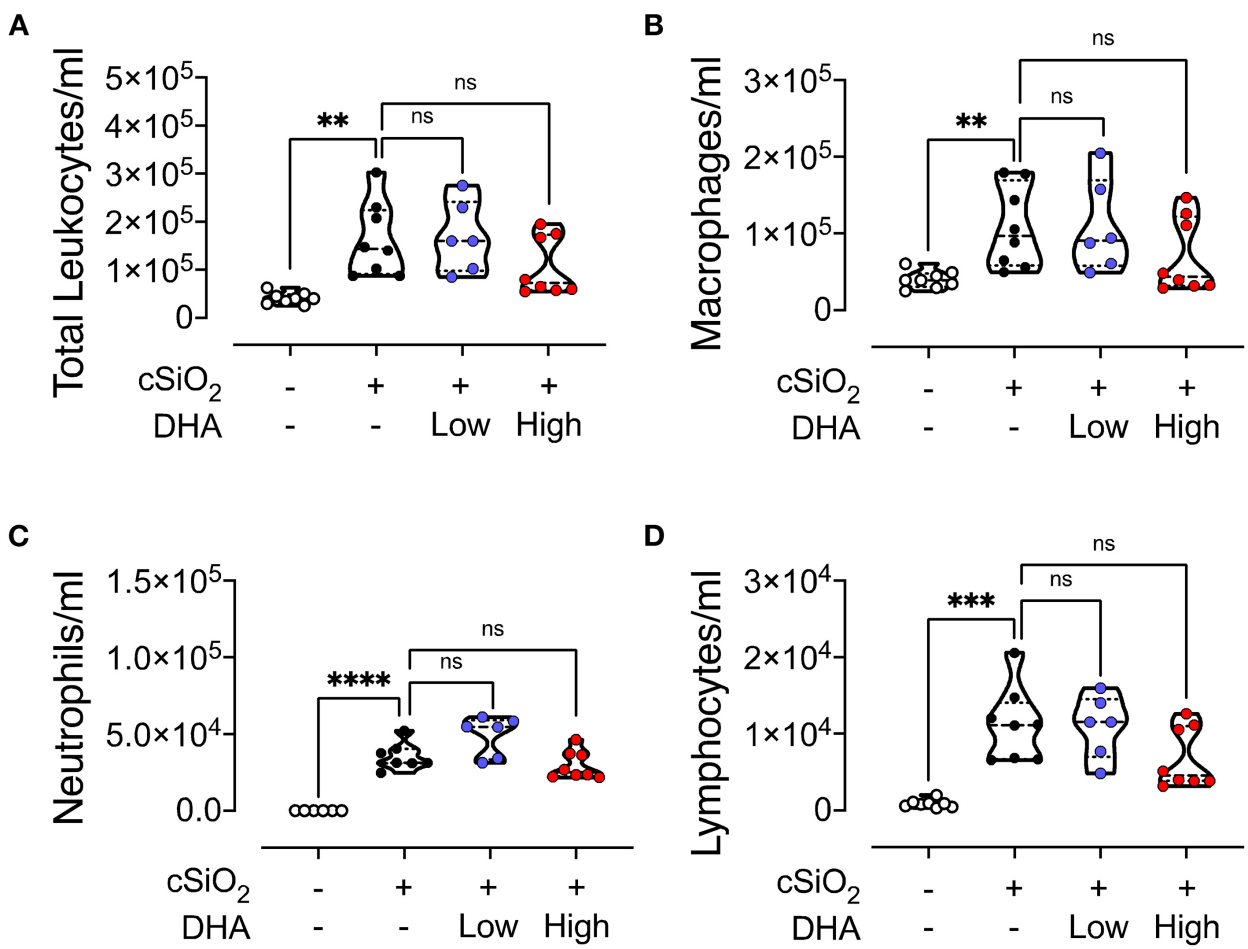
cSiO_2_-induced inflammatory cell recruitment into the lungs is modestly attenuated by DHA feeding. 13 week following the final cSiO_2_ instillation, mice were necropsied and BALF collected. The BALF cell fraction was assessed for **(A)** total cells, **(B)** macrophages, **(C)** neutrophils, and **(D)** lymphocytes as measures of inflammatory cell recruitment. cSiO_2_ significantly increased the number of each cell type in the BALF. Total leukocytes, macrophages, and lymphocytes appeared to be slightly reduced by the high DHA diet, though the difference was not significant. Statistical comparisons were performed as described in the Methods section. ****p ≤ 0.0001, ***p ≤ 0.001, **p ≤ 0.01, ns = not significant.

Intranasal instillation in Cohort 1 mice fed CON diet with cSiO_2_ resulted in significant elevations of total leukocytes, macrophages, lymphocytes, and neutrophils in the BALF compared to vehicle-treated CON-fed mice (Figures 4A–D). These cell populations in cSiO_2_/low DHA and cSiO_2_/high DHA groups did not differ from the cSiO_2_/CON group. cSiO_2_ instillation of CON-fed NZBWF1 mice induced elevations in BALF of the proinflammatory cytokine TNF-α, the chemokine MCP-1, and the B cell stimulation factor BAFF, however, intervention with low or high DHA diets did not affect TNF-α, MCP-1, or BAFF responses (Supplementary Figure 1).

### Lymphoid Cell Recruitment and ELS Development in the Lungs

**Figure 5:**
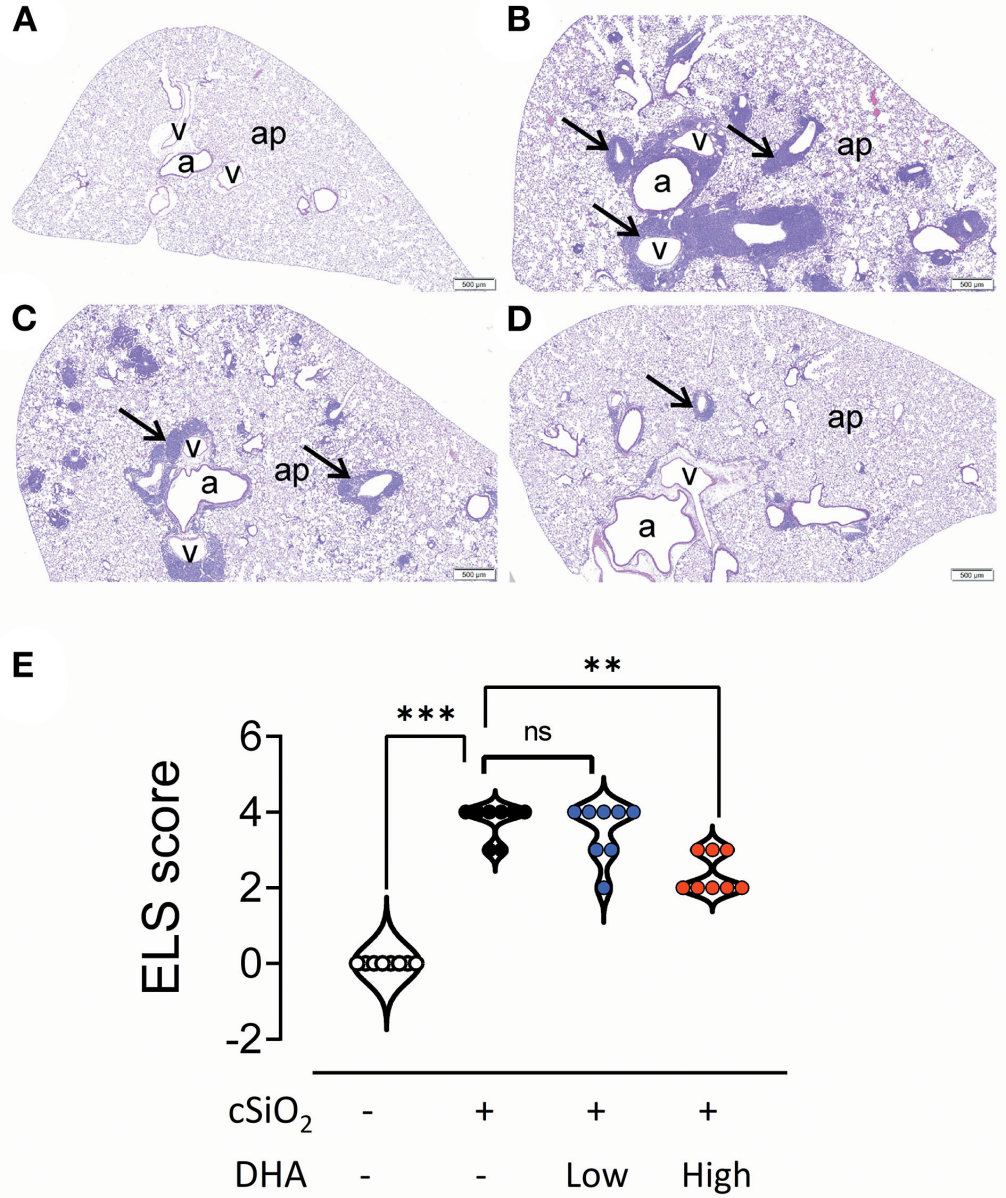
High DHA diet reduces perivascular and peribronchiolar lymphoid aggregates in the lungs of cSiO_2_-treated mice. Light photomicrographs of hematoxylin and eosin-stained lung tissue sections **(A–D)** from mice fed control diet and intranasally instilled with saline vehicle (Veh/CON, **A**), fed control diet and intranasally instilled with cSiO_2_ (cSiO_2_/CON, **B**), fed 0.4 % DHA diet starting one week after the end of the intranasal cSiO_2_ instillations (cSiO_2_/low DHA, **C**), or fed the 1.0% DHA diet starting one week after the end of the intranasal cSiO_2_ instillations (cSiO_2_/high DHA, **D**). Marked perivascular and peribronchiolar airway lymphoid cell accumulation (ectopic lymphoid structures [ELS]; arrows) observed in the lung of cSiO_2_/CON mouse that did not receive DHA treatment **(B)**. Slightly less severe ELS formation around vessels and airways in the lung of cSiO_2_/low DHA mouse **(C)**. Only minimal perivascular and peribronchiolar lymphoid aggregates in the lung of cSiO_2_/high DHA mice **(D)**. b, bronchiolar airway; v, blood vessel, a, alveolar parenchyma. **(E)** Individual sections were semi-quantitatively scored for ELS using the following criteria: (0) no changes compared to control mice; (1) minimal (<10%); (2) slight (10–25%); (3) moderate (26–50%); (4) severe (51–75%); or (5) very severe (>75%) of total area affected. Horizontal lines represent mean ± SEM. Statistical comparisons were performed as described in the Methods section. ***p ≤ 0.001, **p ≤ 0.01, ns = not significant.

**Figure 6:**
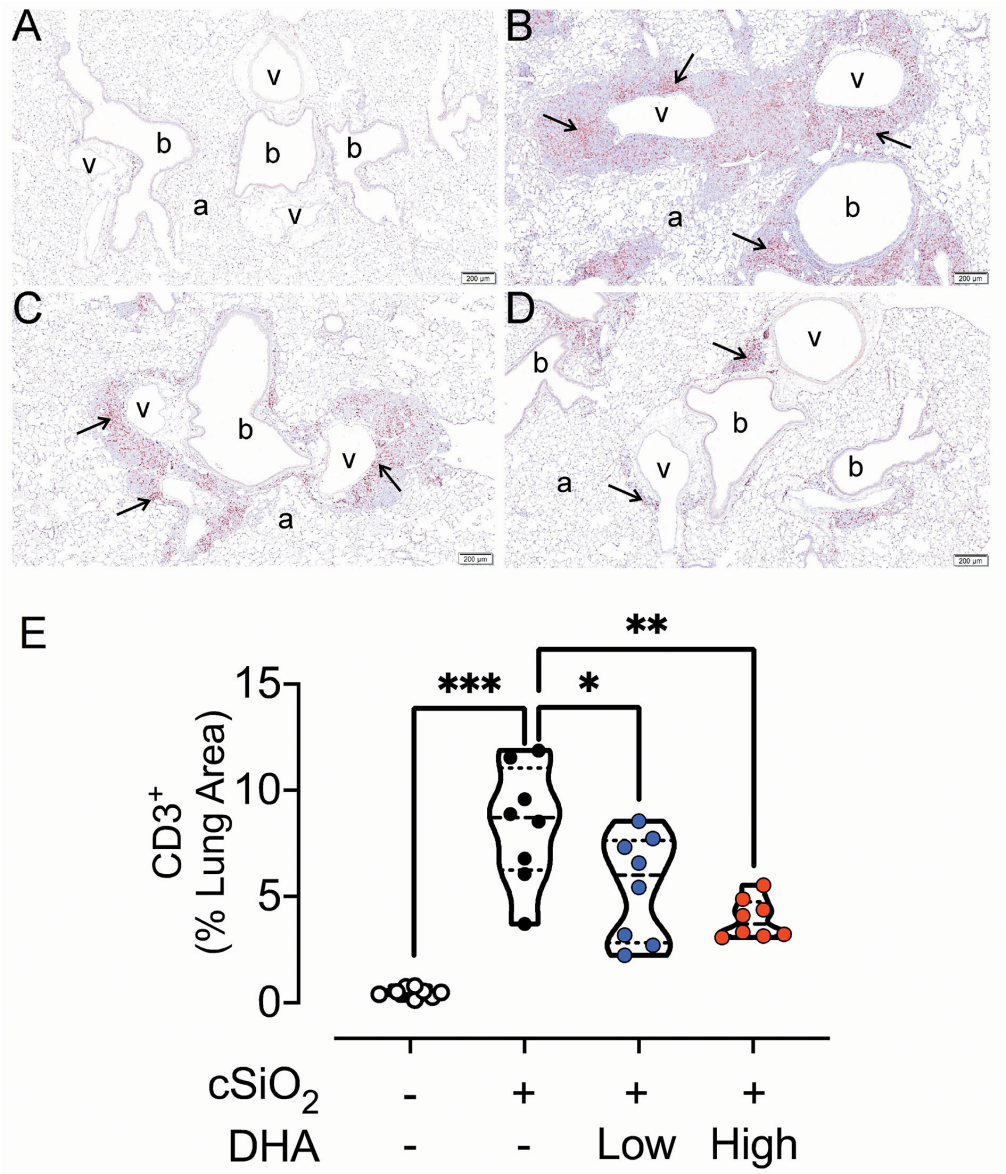
DHA-containing diets reduce CD3^+^ T cell infiltration in cSiO_2_-instilled mice. Light photomicrographs of lung tissue sections immunohistochemically stained for CD3^+^ T cells (arrows) from mice fed control diet and intranasally instilled with saline vehicle Veh/CON, **A**), fed control diet and intranasally instilled with cSiO_2_ (cSiO_2_/CON, **B**), fed 0.4 % DHA diet starting one week after the end of the intranasal cSiO_2_ instillations (cSiO_2_/low DHA, **C**), or fed the 1.0% DHA diet starting one week after the end of the intranasal cSiO_2_ instillations (cSiO_2_/high DHA, **D**). Marked accumulation of perivascular and peribronchiolar CD3^+^ T cells (arrows) in lung of cSiO_2_/CON mouse. Moderate, minimal and no accumulations of CD3^+^ T cells around blood vessels (v) and bronchioles (b) in the lungs of cSiO_2_/low DHA **(C)**, cSiO_2_/high DHA **(D)**, and Veh/CON **(A)** mice, respectively. a, alveolar parenchyma. **(E)** depicts percentage of CD3^+^ lung tissue. Statistical comparisons were performed as described in the Methods section. ***p ≤ 0.001, **p ≤ 0.01, *p ≤ 0.05, ns =not significant.

**Figure 7:**
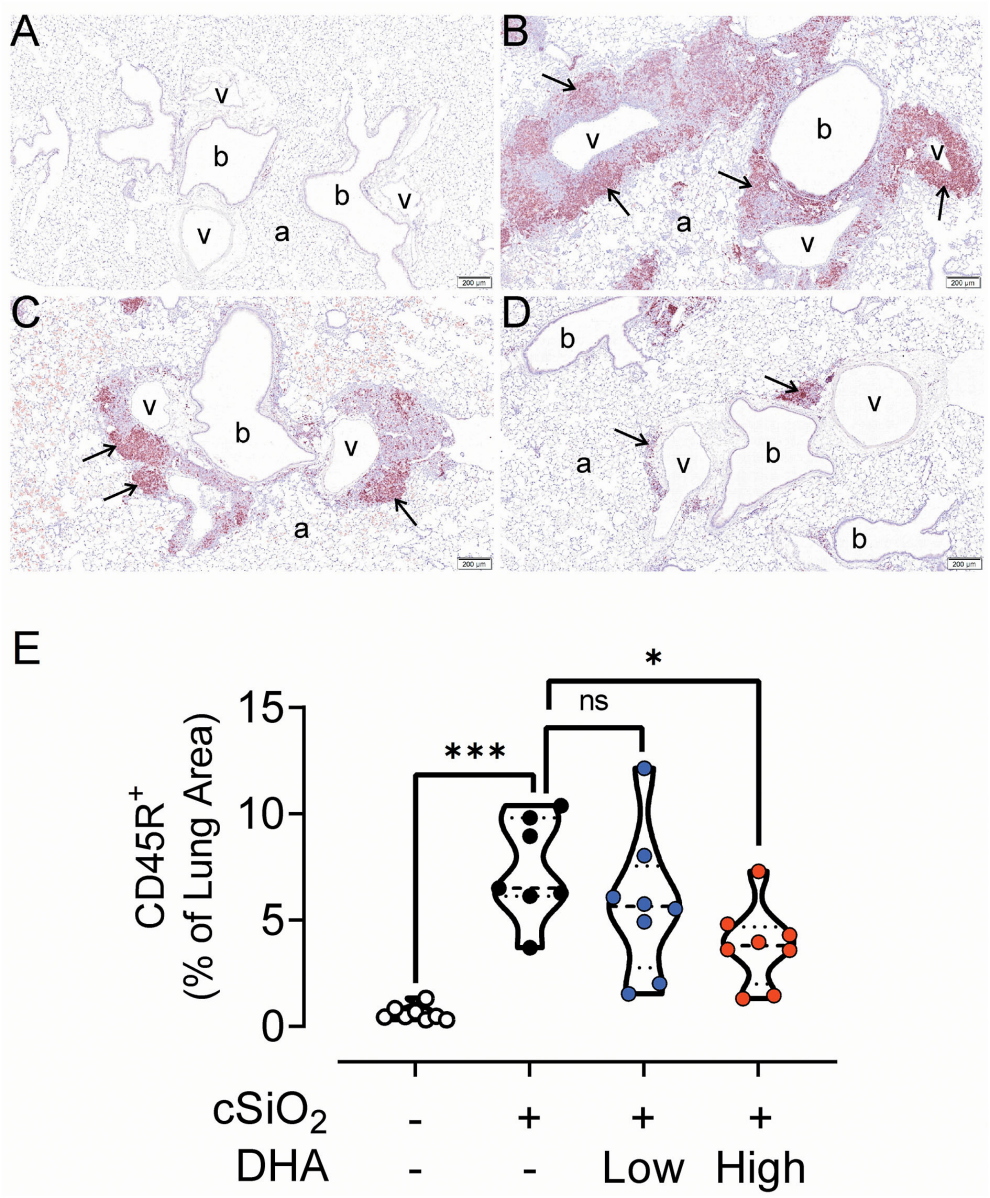
High DHA diet reduces CD45R^+^ B-cell infiltration in the lungs of cSiO_2_-treated mice. Light photomicrographs of lung tissue sections immunohistochemically stained for CD45R^+^ B cells (arrows) from mice fed control diet and intranasally instilled with saline vehicle (Veh/CON, **A**), fed control diet and intranasally instilled with cSiO_2_ (cSiO_2_/CON, **B**), fed 0.4 % DHA diet starting one week after the end of the intranasal cSiO_2_ instillations (cSiO_2_/low DHA, **C**), or fed the 1.0% DHA diet starting one week after the end of the intranasal cSiO_2_ instillations (cSiO_2_/high DHA, **D**). Marked perivascular and peribronchiolar B lymphoid cell accumulations (arrows) in lung of cSiO_2_/CON mouse. Moderate, minimal and no accumulations of B lymphoid cells around blood vessels (v) and bronchioles (b) in cSiO_2_/low DHA **(C)**, cSiO_2_/high DHA **(D)**, and Veh/CON (**A**) mice, respectively. a, alveolar parenchyma. **(E)** depicts percentage of CD45R+ stained lung tissue. Statistical comparisons were performed between groups, as described in the Methods section. ***p ≤ 0.001, *p ≤ 0.05, ns = not significant.

**Figure 8:**
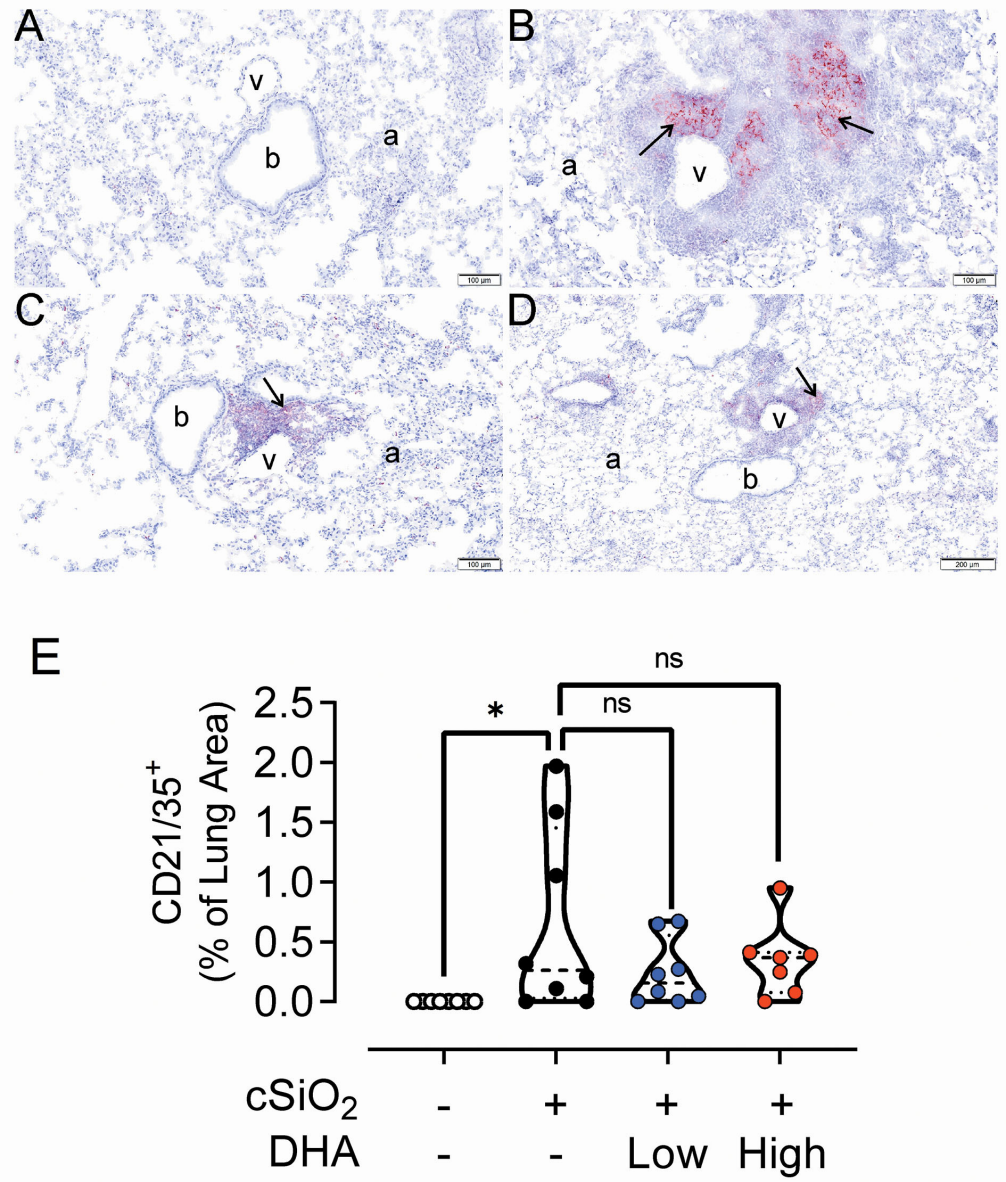
DHA-containing diets reduce CD21/35^+^ follicular dendritic cell (FDC) infiltration in the lungs of cSiO_2_-treated mice. Light photomicrographs of lung tissue sections immunohistochemically stained for CD21/CD35^+^ FDC (arrows) from mice fed control diet and intranasally instilled with saline vehicle (Veh/CON, **A**), fed control diet and intranasally instilled with cSiO_2_ (cSiO_2_/CON, **B**), fed 0.4% DHA diet starting one week after the end of the intranasal cSiO_2_ instillations (cSiO_2_/low DHA, **C**), or fed the 1.0% DHA diet starting one week after the end of the intranasal cSiO_2_ instillations (cSiO_2_/high DHA, **D**). Marked perivascular and peribronchiolar accumulations of follicular dendritic cells in lung of cSiO_2_/CON mouse. Moderate, minimal and no accumulations of follicular dendritic cells around blood vessels (v) and bronchioles (b) in cSiO_2_/low DHA **(C)**, cSiO_2_/high DHA **(D)**, and Veh/CON **(A)** mice, respectively. a, alveolar parenchyma. **(E)** depicts percentage of CD21/35+ stained tissue. Statistical comparisons were performed between groups, as described in the Methods section. *p ≤ 0.05, ns = not significant.

No histopathology was microscopically found in the lungs of Cohort 1 Veh/CON mice (controls) that received intranasal instillations of saline vehicle alone, fed control diet and were sacrificed 13 week after the last instillation (Figure 5A). Lungs from cSiO_2_/CON mice that were similarly fed and sacrificed at 13 week PI had the most severe pulmonary lesions due to cSiO_2_ (Figure 5B). These lesions were most prominent in the hilar region of the lung lobe. cSiO_2_-induced histopathology consisted of conspicuous mononuclear lymphoid cell accumulations in the interstitial space surrounding small- and large-diameter blood vessels (arterial and venous) and to a lesser extent in the interstitium around bronchiolar airways (Figure 5B).These perivascular and peribronchiolar accumulations contained CD3^+^ T cells (Figures 6A,B), CD45R^+^ B cells (Figures 7A,B), CD21^+^/CD34^+^ FDC (Figures 8A,B) that together were morphologically consistent with ELS formation. In addition to ELS in these cSiO_2_-instilled mice, there were adjacent areas of alveolitis characterized by alveolar infiltration of a mixed inflammatory cell population of neutrophils, monocytes/macrophages, and lymphocytes along with alveolar airspace accumulation of amorphous proteinaceous material (proteinosis), cellular/nuclear debris, and birefringent cSiO_2_ particles within and outside of vacuolated alveolar macrophages. cSiO_2_-induced lung lesions, especially ELS, were similar in cellular and distributional character in cSiO_2_/low DHA and cSiO_2_/CON mice (Figures 5B,C,E), but markedly less severe in cSiO_2_/high DHA mice (Figures 5D,E). Consumption of high but not low DHA diet similarly reduced perivascular and peribronchiolar accumulations containing CD3^+^ T cells (Figures 6C–E) and CD45R^+^ B cells (Figures 7C–E) with a similar trend being evident for CD21^+^/CD34^+^ FDC (Figures 8C–E). The severity of alveolar proteinosis and accumulation of cellular/debris and birefringent cSiO_2_ particles, however, were not noticeably different among the lungs of cSiO_2_/CON, cSiO_2_/low DHA, and cSiO_2_/high DHA mice (Figures 5B–D).

### mRNA Signatures in the Lungs

Repeated cSiO_2_ exposure has been previously shown to induce expression of numerous inflammation-, chemokine-, and IFN-associated genes (42) and, furthermore, these responses can be suppressed by prophylactically feeding low and high DHA diets (25). In distinction from that preventive study, we found in Cohort 1 that DHA intervention after the insult did not impact expression of cSiO_2_-triggered mRNA signatures associated with inflammation, chemokine production, or IFN signaling (Supplementary Figure 2).

### IgG AAb Responses in Plasma and BALF

**Figure 9:**
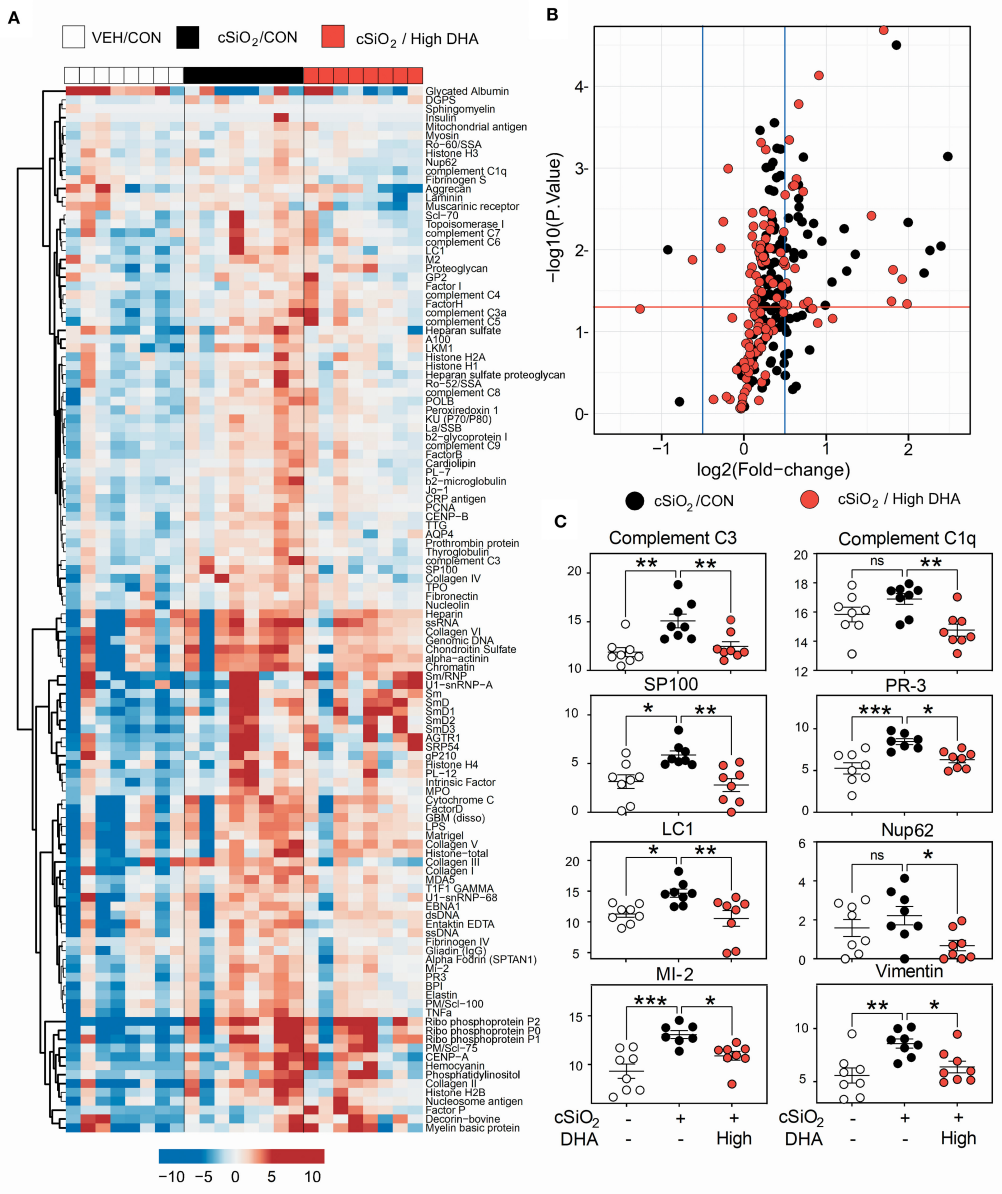
High DHA supplementation inhibits a broad spectrum of IgG autoantibodies induced by cSiO_2_. **(A)** Heat maps with unsupervised clustering (Euclidian distance method) of 122 AAbs depict Ab-score values for IgG expression in BALF. White, black, and red in the top key indicates the Veh/CON, cSiO_2_/CON, and cSiO_2_/high DHA experimental groups, respectively. Scale bar values reflect the range of variance-stabilized Ab scores, which were centered across rows. **(B)** Volcano plots depicting cSiO_2_-induced IgG AAb responses in plasma at 13 week post final exposure in lung of cSiO_2_-treated NZBWF1 mice. The individual AAg within each treatment group are plotted as log_2_ (fold change) vs. -log_10_ (*p*-value). Black dots indicate cSiO_2_/CON relative to Veh/CON treated mice; red dots indicate cSiO_2_/high DHA relative to Veh/Con. The vertical blue lines represent the cut-off levels log_2_ fold change < −0.5 or >0.5. The horizontal red line represents the cut-off levels *p*-values ≥ 0.05 (log10 P≥ 1.2). **(C)** DHA intake suppressed cSiO_2_-induced IgG AAb responses against selected AAgs. Data presented as mean ± SEM. Statistical comparisons were performed as described in the Methods section. ***p ≤ 0.001, **p ≤ 0.01, *p ≤ 0.05, ns = not significant.

A vast repertoire of IgG AAbs associated with lupus and other autoimmune diseases was elicited in plasma of Cohort 1 mice following cSiO_2_ treatment, many of which were suppressed by high DHA supplementation (Figures 9A,B). These included anti-complement 3 (lupus), anti-complement C1q (lupus), anti-SP100 (lupus), PR-3 (vasculitis), anti-LC-1 (autoimmune hepatitis), anti-Nup62 (primary biliary cirrhosis), anti-MI-2 (myositis), and anti-vimentin (rheumatoid arthritis) (Figure 9C). Although cSiO_2_ likewise induced a diverse array of IgG AAbs in BALF, these were largely unaffected by feeding DHA (Supplementary Figure 3).

### Glomerulonephritis and T Cell Infiltration in the Kidney

**Figure 10:**
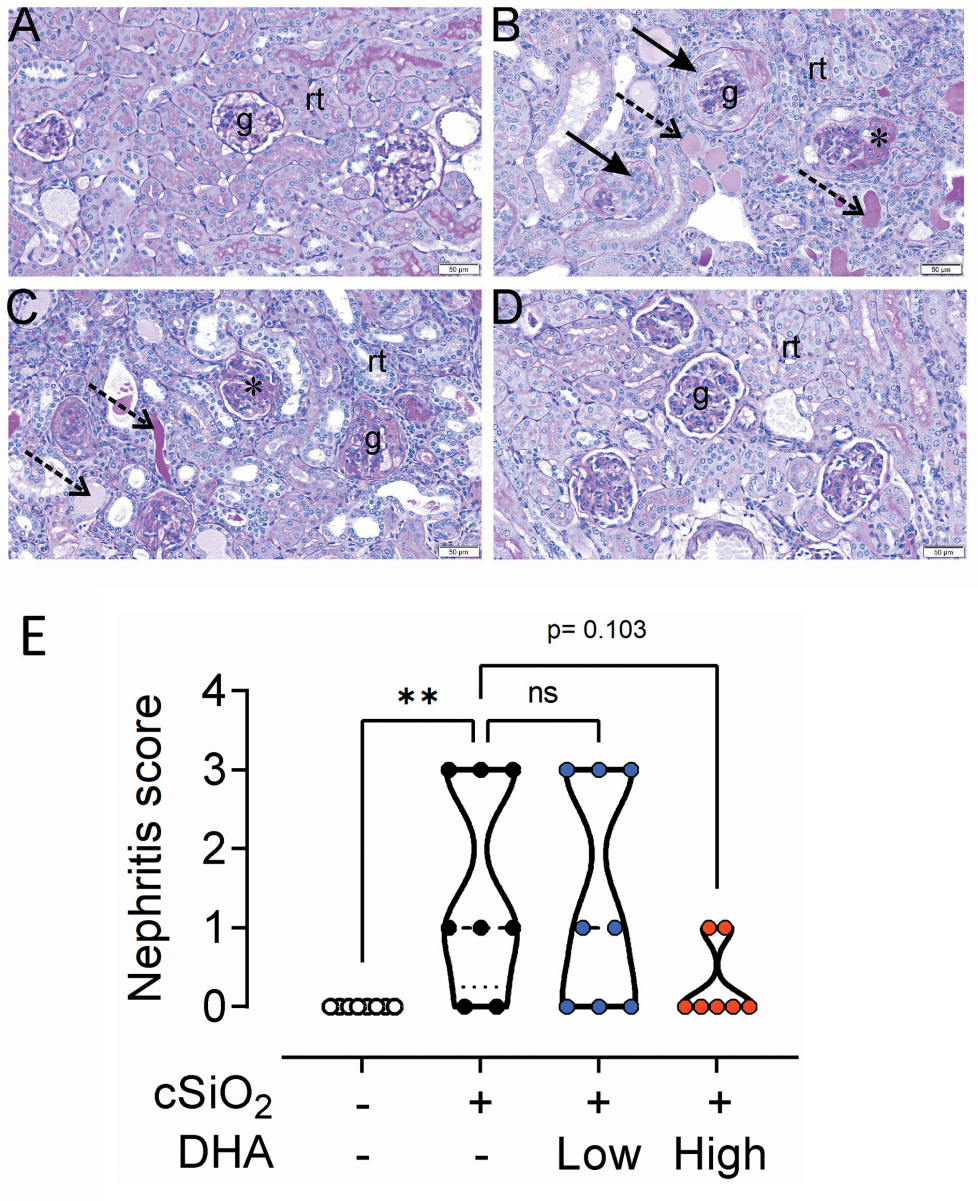
High DHA diet slightly inhibits cSiO_2_-triggered glomerulonephritis. Representative periodic acid Schiff-stained kidney tissue sections **(A–D)** from mice fed control diet and intranasally instilled with saline vehicle (Veh/CON; **A**), fed control diet and intranasally instilled with cSiO_2_ (cSiO_2_/CON; **B**), fed 0.4% DHA diet starting one week after the end of the intranasal cSiO_2_ instillations (cSiO_2_/low DHA; **C**), or fed 1.0% DHA diet starting one week after the end of the intranasal cSiO_2_ instillations (cSiO_2_/high DHA; **D**). Kidney histopathology consisting of shrunken glomerular tufts, mesangial matrix expansion, in glomeruli (asterisk), crescent-shaped masses of proliferating cells in Bowman's space (solid arrow), regenerative tubular epithelium, and tubular protein accumulation (dotted arrows) were evident only in cSiO_2_-instilled mice that received no or low DHA treatment **(D)**. No renal histopathology was present in Veh/CON or cSiO_2_/high DHA treatment. g, glomerulus; rt, cortical renal tubules. **(E)** Semiquantitative scoring based on histopathology conducted as described in Materials and Methods. **p ≤ 0.01, ns = not significant.

No histopathology was present in the kidneys of Veh/CON mice (Figure 10A). Moderate to marked membranoproliferative glomerulonephritis was evident in the cSiO_2_/CON group (Figure 10B). This was characterized by thickening of PAS^+^ interstitial tissue in glomeruli, hyperplasia of glomerular mesangial cells, parietal epithelial hyperplasia in Bowman's capsule (crescent formation), mixed inflammatory cell infiltration in the renal cortex (neutrophils and mononuclear cells), regenerative hyperplasia of renal tubular epithelium, and amorphous accumulation of proteinaceous and hyaline material in dilated renal tubules. Similar histopathology but of slightly less severity was evident in the kidneys of cSiO_2_/low DHA mice (Figure 10C). Minimal to no renal histopathology was present in cSiO_2_/high DHA mice (Figure 10D). Semiquantitative scoring indicated that there was a trend toward inhibition of cSiO_2_ induced glomerulonephritis by high DHA supplementation (Figure 10E).

**Figure 11:**
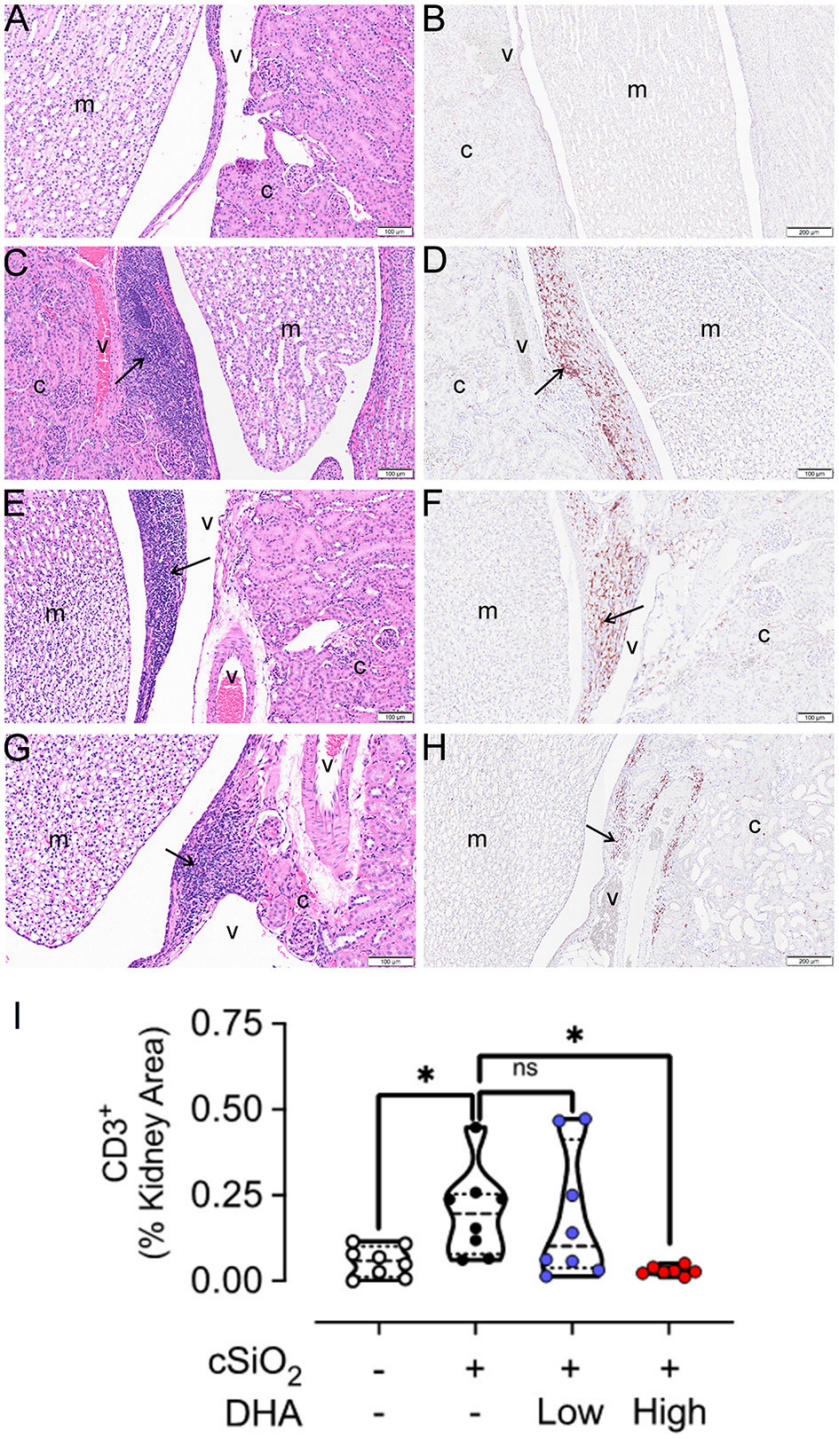
High DHA diet suppresses cSiO_2_-induced CD3^+^ T cell infiltration into the kidney. Light photomicrographs of hematoxylin and eosin-stained renal tissue sections **(A,C,E,G)** from the hilar region and light photomicrographs of kidney tissue sections immunohistochemically stained (arrows) for CD3^+^ T cells **(B,D,F,H)**. Kidneys taken from mice at 13 weeks after the final intranasal saline intranasal instillation and fed control diet (Veh/CON, **A,B**), cSiO_2_ instillation and fed control diet (cSiO_2_/CON, **C,D**), cSiO_2_ instillation and fed 0.4% DHA diet (cSiO_2_/low DHA, **E,F**), and cSiO_2_ instillation and fed 1.0% DHA diet (cSiO_2_/high DHA, **G,H**).Lymphoid cell accumulations (arrows) in the interstitial tissue adjacent to interlobular blood vessels (v) in the renal cortex (c) **(C,E,G)**). Marked perivascular accumulation of lymphoid cells in the renal cortex (c) from cSiO_2_/CON mouse **(C)**. Moderate, mild and no perivascular accumulations of lymphoid cells in the kidneys of cSiO_2_/low DHA **(E)**, cSiO_2_/high DHA **(G)**, and Veh/CON **(A)** mice, respectively. Marked perivascular accumulation of CD3^+^ T cells (arrows) in kidney of cSiO_2_/CON mouse **(D)**. Moderate, minimal and no perivascular accumulations of T lymphoid cells in the kidneys of cSiO_2_/low DHA **(F)**, cSiO_2_/high DHA **(H)**, and Veh/CON **(B)** mice, respectively. m, medulla of the renal papilla that extends into the renal pelvis. **(I)** depicts the percentage of CD3^+^ renal tissue. Statistical comparisons were performed as described in the Methods section. *p ≤ 0.05, ns = not significant.

Cohort 1 kidneys were further subjected to cellularity assessment in H&E-stained sections (Figures 11A,C,E,G) and immunohistochemical localization of CD3^+^ T cells (Figures 11B,D,H,I). Accumulations of renal lymphoid cells (Figure 11A) or CD3^+^ cells (Figure 11B) were not evident in Veh/CON mice. However, in the renal cortex at the hilar region of the kidney of Veh/cSiO_2_ mice, there were mononuclear lymphoid cell accumulations resembling ELS in interstitial tissue adjacent to interlobular blood vessels (arteries and veins) and the renal pelvis (Figure 11C). These increases corresponded with elevated CD3^+^ cells (Figure 11D). There were less lymphoid and CD3^+^ T cells in cSiO_2_/low DHA mice (Figures 11E,F) with decreases being more marked in cSiO_2_/high DHA mice (Figures 11G,H). Morphometry confirmed that cSiO_2_ treatment induced the accumulation of CD3^+^ T cells, which was inhibited by feeding high DHA diet (Figure 11I).

### Proteinuria and Survival

**Figure 12:**
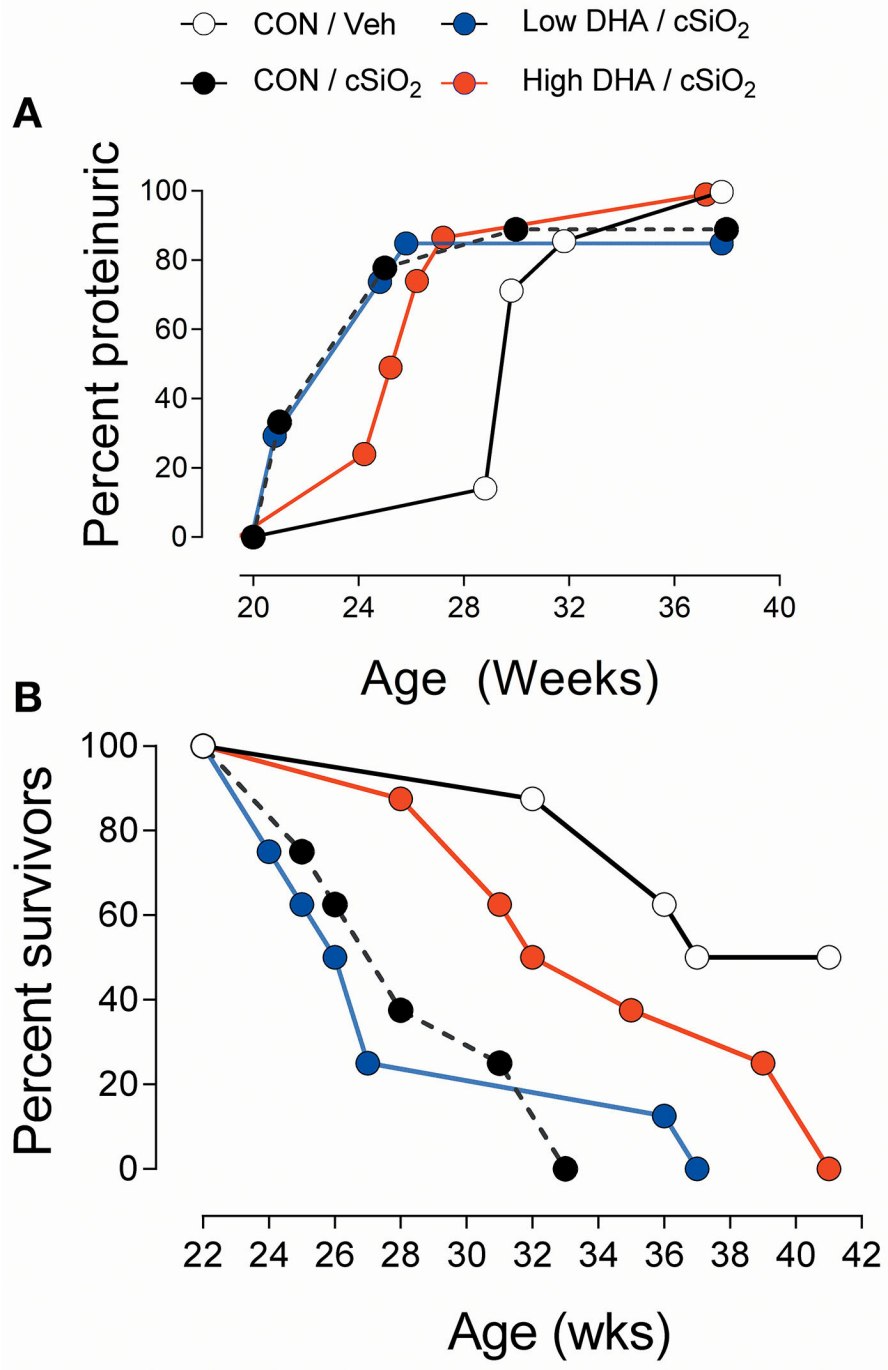
High DHA diet delays cSiO_2_-triggered onset of proteinuria and prolongs survival in cSiO_2_-treated lupus-prone NZBWF1 mice. Animals in Cohort 2 were initiated on control diets 1 week following the final cSiO_2_ instillation and animals monitored for proteinuria (≥300 mg/dl) using clinical dipsticks **(A)** and for survival using moribund criteria **(B)**.

The kidney histopathology in Cohort 1 mice was highly predictive of the development of proteinuria in Cohort 2 mice (Figure 12A). Proteinuria onset occurred at age 21 week in cSiO_2_/CON and cSiO_2_/low DHA groups, whereas proteinuria was first evident at 25 and 29 week age in cSiO_2_/high DHA and Veh/CON groups, respectively. Further consonant with glomerulonephritis findings, Kaplan-Meier analysis of Cohort 2 revealed that median survival times for cSiO_2_/CON, cSiO_2_/low DHA, cSiO_2_/high DHA, and Veh/CON groups were 28, 27, 34, and 39 week, respectively (Figure 12B). Thus, consumption of high DHA diet suppressed proteinuria and extended survival of cSiO_2_-treated mice.

## Discussion

While an individual's genome is a critical determinant of predisposition toward autoimmunity, a multitude of lifestyle and environmental factors can potentiate or attenuate disease penetrance. Here, we investigated for the first time how dietary supplementation with the ω-3 PUFA DHA influences progression of autoimmunity initiated in NZBWF1 mice by repeated intranasal exposures to the autoimmune trigger cSiO_2_. DHA consumption was found to dose-dependently increase DHA, and to a lesser extent, EPA, in the plasma, lung, and kidney. Supplementation with low or high DHA did not impact the inflammatory state of the lung as represented both by leukocyte numbers/profiles and cytokine concentrations in BALF and by expression of proinflammatory, chemokine, or IFN-regulated genes. However, several novel findings suggest that DHA markedly reduced progression of preexistent autoimmunity in the lung and that this ultimately impacted the systemic and renal compartments. First, provision of high DHA diet attenuated cSiO_2_-induced T**-** and B cell recruitment and ELS development in the lung. Second, high DHA supplementation suppressed cSiO_2_-triggered elevations of a wide spectrum of pathogenic AAbs in plasma. Third, mice consuming high DHA exhibited delayed onset and progression of glomerulonephritis and proteinuria. Fourth, lifespans of cSiO_2_-instilled mice were extended in those fed high DHA as compared to those fed control diets. Finally, amelioration of cSiO_2_ -triggered autoimmune effects and reduction in survival time were not observed in mice fed low DHA diet. Cumulatively, the results of this preclinical study support the contention that DHA supplementation at 5 g/d HED was an effective therapeutic intervention for slowing progression of established autoimmunity induced by the environmental toxicant cSiO_2_.

Since inhaled cSiO_2_ is inefficiently cleared from the lung, the long-term persistence of these particles causes a perpetual cycle in alveolar macrophages that includes: (i) phagocytosis, (ii) lysosomal membrane permeabilization, (iii) inflammasome activation, (iv) cell death, and (v) cSiO_2_ release. Additional rounds of this cycle likely promote chronic unresolved inflammation in the pulmonary compartment, thereby driving the loss of immunological tolerance (43, 44). Consistent with this paradigm, intranasal instillation of NZBWF1 mice with cSiO_2_ causes sterile inflammation in the lung characterized by leukocyte recruitment, elevated cytokines and chemokines, upregulated mRNA signatures associated with chemokines, cytokines, IFN, complement, and adhesion molecules (42). Our previous studies show that preventive feeding of NZBWF1 mice with low or high DHA diets before cSiO_2_ instillation impairs inflammatory recruitment of macrophages, neutrophils, and lymphocytes into the lung alveolar space at 9 and 13 week PI (13). While consumption of high but not low DHA diets suppressed cSiO_2_-induced TNF-α, MCP-1, and BAFF in BALF (30), both levels inhibit cSiO_2_-triggered inflammatory/autoimmune gene expression at 1, 5, and 9 week PI, and to a lesser extent, at 13 week PI (25). Unlike these previously reported preventive studies, we observed here in Cohort 1 that when initiated after cSiO_2_ treatment, neither dietary DHA level affected the overall inflammatory state of the lung relative to cell numbers, leukocyte profiles, cytokine concentrations, or gene expression. Intriguingly and consistent with earlier preventive studies (13, 30), high DHA consumption attenuated development of T-cell**-** and B-cell-containing ELS in the lung. One explanation for these dichotomous responses is that in the therapeutic regimen, DHA might slow inflammation at earlier time points thereby attenuating downstream ectopic lymphoid neogenesis, however, by 13 week PI, this attenuation was no longer apparent because the cSiO_2_-driven vicious cycle of cell death and inflammation overwhelms the pro-resolving effects of ω-3 PUFAs. Nevertheless, suppression of ectopic lymphoid neogenesis was still observable. Therefore, in future investigations evaluating the therapeutic efficacy of ω-3 PUFAs in this and other preclinical models of autoimmunity, it will be important to measure inflammatory biomarkers at multiple time points and relate these to developing autoimmunity.

Because cSiO_2_ induces cell death (45–47) and impedes efferocytosis (48), resultant dead cell accumulation and secondary necrosis likely unmasks a rich and diverse autoantigen profile in the lung that induces a broad range of AAbs. Consistent with this possibility, repeated intranasal cSiO_2_ instillation in NZBWF1 mice a diverse repertoire of AAbs in BALF and plasma that could be delayed by preventive low and high DHA supplementation (26, 30). In the present therapeutic study, consumption of high DHA diet after cSiO_2_ treatment also suppressed elevation of a large repertoire of pathogenic IgG AAbs in the plasma of Cohort 1 that bound autoantigens that included C1q, C3, SP100, PR-3, LC1, Nup62, MI-2, and vimentin. Interestingly, feeding either DHA diet did not affect IgG AAb responses in the BALF. A potential reason for the observed differences between DHA's effects in the systemic and pulmonary compartments is that plasma IgG AAbs might arise from B-cells that have migrated from the lung to the spleen and other lymph nodes and that increasing the membrane ω-3 PUFA content might interfere with this homing. Relatedly, Cucchi et al. (49) recently reported that EPA and DHA influence CD4^+^ T cell motility and alter their capacity to reach target tissues by interfering with the cytoskeletal changes necessary for cell migration. Clearly, further research is needed to address whether ω-3 PUFAs similarly influence B cell motility.

Consistent with histopathological indications of glomerulonephritis and renal accumulation of T-cells in Cohort 1, mice consuming high DHA diet exhibited delayed onset and progression of proteinuria in Cohort 2. These findings are significant because glomerulonephritis is a frequent and serious organ-specific manifestation of lupus, with 10 to 30% of patients developing kidney failure (50). Various T cell subsets can activate the glomerular immune response by releasing nephritogenic cytokines or cooperating with B-cells, macrophages, and dendritic cells (51). The observation in Cohort 1 that mice fed high DHA diet after cSiO_2_ exposure exhibited extended lifespans compared to CON likely relates to reduced glomerulonephritis.

American diets contain many times more ω-6 PUFAs than ω-3 PUFAs, thereby skewing tissue phospholipid fatty acid content profoundly toward ω-6 PUFAs (52, 53). At the mechanistic level, increasing the ratio of the ω-3 PUFAs DHA and EPA to the ω-6 PUFA ARA in tissues can influence inflammation and autoimmune pathogenesis in numerous ways. When incorporated into the cell membrane, ω-3 PUFAs impede lipid raft formation and inhibit activation of transmembrane receptors linked to innate and adaptive immune processes (54). Intracellular and extracellular phospholipases can liberate DHA and EPA from the membrane (55, 56), enabling them to activate transmembrane receptors that interfere with proinflammatory signaling pathways (57, 58). Additionally, ω-3 PUFAs are known ligands for PPARγ, a transcription factor that can hamper NF-kB-dependent transcription of genes associated with inflammation and autoimmunity (59, 60). Furthermore, ω-6 PUFAs, including ARA, are metabolized to proinflammatory prostaglandins, thromboxanes, and leukotrienes or the pro-resolution lipoxins (61). As shown here, dietary DHA supplementation dose-dependently decreases the ARA content in plasma, lung, and kidney, thereby reducing the availability of this key substrate for generation of proinflammatory mediators.

DHA and EPA also directly compete with ω-6 PUFAs as enzymatic substrates to specialized pro-resolving mediators (SPMs) (e.g., resolvins, protectins, maresins) (62) that can impede inflammatory signaling (63, 64) and promote efferocytosis which are critical to maintaining immune tolerance (63, 64). Germaine to the present study, resolvin D1 and resolvin D2 and maresin 1 can (i) suppress cytokine production by activated CD8^+^ T cells and CD4^+^ T helper 1 (TH1) and TH17 cells, (ii) inhibit naïve CD4^+^ T cell differentiation into TH1 and TH17 cells by GPR32 and ALX/FPR2 receptor-mediated down-regulation of critical transcription factors, and (iii) enhance *de novo* generation and function of Foxp3^+^ regulatory T (Treg) cell (65). Furthermore, Crouch et al. (66) reported that in obese mice the DHA-derived SPMs 14-HDHA and 17-HDHA control B cell numbers and lower circulating IgG2c. Thus, it is tempting to speculate that in the lupus model system, the effects of dietary DHA may be driven part by the capacity of this novel family of molecules to regulate T cell subset number, B cell subset numbers, and proinflammatory antibody levels. Altogether, altering the tissue balance by increasing ω-3 PUFAs and decreasing ω-6 PUFAs likely promotes resolution over inflammation, thus preserving immunological tolerance.

As shown here, low and high DHA diets dose-dependently altered relative tissue PUFA content by increasing ω-3 PUFAs and decreasing ω-6 PUFAs, most notably ARA. We recently performed an analysis relating the red blood cell (RBC) ω-3 PUFA levels to disease severity and progression in previous studies we had performed on NZBWF1 mice supplemented with DHA on the background of three unique diets (15). Most autoimmune and inflammatory endpoints, including inflammatory gene expression, cytokine concentrations, immune cell infiltration, and AAB production negatively correlated with RBC omega-3 levels. Since these prior studies exclusively utilized prophylactic DHA supplementation, animals already had ω-3 PUFA-enriched membranes at the time of cSiO_2_ instillation and induction of disease flaring. In the present study, therapeutic administration of the low DHA dose did not effectively suppress lupus flaring, even though this dose provided in this study achieved similar RBC ω-3 PUFA levels shown to confer moderate protection in the prophylactic supplementation model. Rather, only mice fed the high DHA diet after cSiO_2_ showed reduced progression of ectopic lymphoid neogenesis, systemic autoimmunity, and glomerulonephritis. From a clinical perspective, this would suggest that slowing progression of established autoimmunity requires higher ω-3 PUFA intake—and subsequently, higher RBC ω-3 PUFA content—than does preventing onset of autoimmunity.

The high DHA diets equate to an HED of 5 g/d which is a realistic and safe human dose (67).Consistent with the observation here that attenuating effects were only associated with the high DHA dose, human investigations demonstrating marine ω-3 PUFA ameliorative effects on inflammatory mediators or inflammatory cell function typically have employed intakes of > 2 g/d (18). A recent meta-analysis concluded that more than 3 g/d in human clinical trials may be beneficial for lupus (23). Likewise, clinical benefits have been reported in patients with rheumatoid arthritis when consuming approximately ≥ 3.5 g/d of marine ω-3 PUFAs (18). It is therefore pertinent to ask to whether these high intakes can be achieved through diet or require taking supplements. Certain marine algae proficiently elongate shorter chain ω-3 fatty acids to DHA and EPA that make them the primary producers in the food web. Fatty fish such as mackerel and salmon consume these algae and bioconcentrate ω-3 PUFAs, and are the principal source for these fatty acids in the human diet. While a meal of oily fish such as salmon can provide 1.5 to 3 g of marine ω-3 PUFAs, consumers of the typical Western diet do not eat seafood daily. Rather, marine ω-3 PUFA consumption ranges in the tens to low hundreds of mg/d as reviewed by Calder (68). Consistent with this premise, Cave et al. recently assessed EPA and DHA intake in the U.S. according to ethnicity, education, and income using 2003–2014 NHANES data and found that overall, EPA+DHA intake was low, with an average daily intake of 100 mg (69). ω-3 PUFA deficiency can be corrected and DHA and EPA incorporation into tissue phospholipids increased by consuming dietary supplements made from fish or microalgal oil (70). Accordingly, to attain the tissue concentrations of DHA and EPA associated with delayed progression of autoimmunity in the present preclinical study, a patient with lupus would likely need to consume dietary supplements containing marine ω-3 PUFAs.

Strengths of this study included the use of a widely employed lupus-prone mouse model, an established human autoimmune trigger, and physiologically relevant doses of DHA. There were, however, some limitations that will require extra attention in future studies. Since it is possible that DHA supplementation affected pulmonary inflammation and AAb responses in Cohort 1 earlier than 13 week PI, the inclusion of earlier timepoints would have been useful to better understand the temporal effects of this intervention. Additionally, since DHA consumption was found to dose-dependently increase DHA, and to a lesser extent, EPA, in the plasma, lung, and kidney, it is difficult to know the extent to which this latter ω-3 PUFA contributes to amelioration of the autoimmune response. This can be addressed in the future by conducting analogous studies on the effects of EPA supplementation on cSiO2-triggered murine lupus. Also, while gene expression was assessed in the lung, measuring mRNA expression in the spleen and kidney could further expand our understanding of how DHA influenced inflammatory and autoimmune gene signatures in the systemic and renal compartments, respectively. Lastly, characterization of immune/inflammatory cell populations in the lung and kidney using additional immunohistochemical markers for T and B cell subsets, flow cytometry, and/or single cell RNA sequencing could provide further insight into how DHA intervention affected lymphoid and myeloid cells involved in inflammation and autoimmunity.

Taken together, in addition to its previously reported preventive effects of DHA supplementation against cSiO_2_ **-**triggered lupus in NZBWF1, the present therapeutic dietary regimen offers promise for suppressing progression established autoimmunity and minimizing the number and severity of lupus flares following respiratory exposures to this commonly encountered occupational dust, or other inhaled environmental toxicants.

## Data Availability Statement

The raw data supporting the conclusions of this article will be made available by the authors, without undue reservation.

## Ethics Statement

The animal study was reviewed and approved by the Institutional Animal Care and Use Committee at Michigan State University (AUF #01/15-021-00).

## Author Contributions

JP: study design, coordination, oversight, funding acquisition, manuscript preparation, and submission. PA: study design, feeding study, necropsy, immunohistochemical analyses and morphometry, and manuscript preparation. KW and LR: data curation, data analysis/interpretation, figure preparation, and manuscript writing. MB: study design, animal handling, feeding study, funding acquisition, and manuscript editing. KG: animal handling, feeding study, necropsy, lab analyses, and manuscript editing. JW: study design, necropsy, and lab analyses. RL: necropsy and lab analyses. PC: gene expression and manuscript editing. AL: fatty acid analysis and interpretation. Q-ZL: microarray, data analysis, and manuscript preparation. JH: study design, lung/kidney histopathology, morphometry, and data analyses. All authors contributed to the article and approved the submitted version.

## Conflict of Interest

The authors declare that the research was conducted in the absence of any commercial or financial relationships that could be construed as a potential conflict of interest.
