# Supplementary material for: Omega-3 Polyunsaturated Fatty Acid Intervention Against Established Autoimmunity in a Murine Model of Toxicant-Triggered Lupus
Source: Front Immunol. 2021 Apr 7;12:653464. doi: 10.3389/fimmu.2021.653464 (PMC8058219; doi:10.3389/fimmu.2021.653464)
Supplement: Supplementary file 1 [file Data_Sheet_1.docx]

Supplementary Material

Omega-3 Polyunsaturated Fatty Acid Intervention Against Established Autoimmunity in a Murine Model of Toxicant-Triggered Lupus

**James J. Pestka^1,2,3^, Peyman Akbari^2,3,4^, Kathryn A. Wierenga ^3,5^, Melissa A. Bates^2,3^, Kristen N. Gilley^2^, James G. Wagner^3,4^, Ryan P. Lewandowski^4^, Lichchavi D. Rajasinghe****^2,3^, Preeti S. Chauhan^2,3^, Adam L. Lock^6^, Quan-Zhen Li^7^, and Jack R. Harkema^3,4^**

**Figures**

- **Supplementary Figure 1.** **cSiO_2_-induced cytokine secretion in the BALF of NZBWF1 mice is not affected by low or high DHA diets.** Panels show secretion of **(A)** TNF-α, **(B)** MCP-1, and **(C)** BAFF. Data are presented as mean ± SEM (n=8).
- **Supplementary Figure 2.** **cSiO_2_-induced gene expression in the lungs of NZBWF1 mice is not affected by low or high DHA diets.** Panels show fold change expression of **(A)** inflammatory, **(B)** chemokine, **(C)** interferon-related genes. Data are presented as mean ± SEM (n=8). *** p<.001, ** p<.01, *, p<0.05.
- Supplemental Figure 3. Effects of DHA intake on cSiO_2_-induced IgG AAb responses in BALF. Heat maps with unsupervised clustering (Euclidian distance method) depict Ab-score values for IgA AAbs for 122 AAgs. White, black, and red in the top bar indicates the VEH/ CON, cSiO_2_/ CON, and cSiO_2_/ High DHA groups. Scale bar values reflect the range of variance-stabilized Ab scores, which were centered across rows.

**Tables**

- **Supplementary Table 1.** Lung fatty acid content of experimental groups
- **Supplementary Table 2.** Kidney fatty acid content of experimental groups


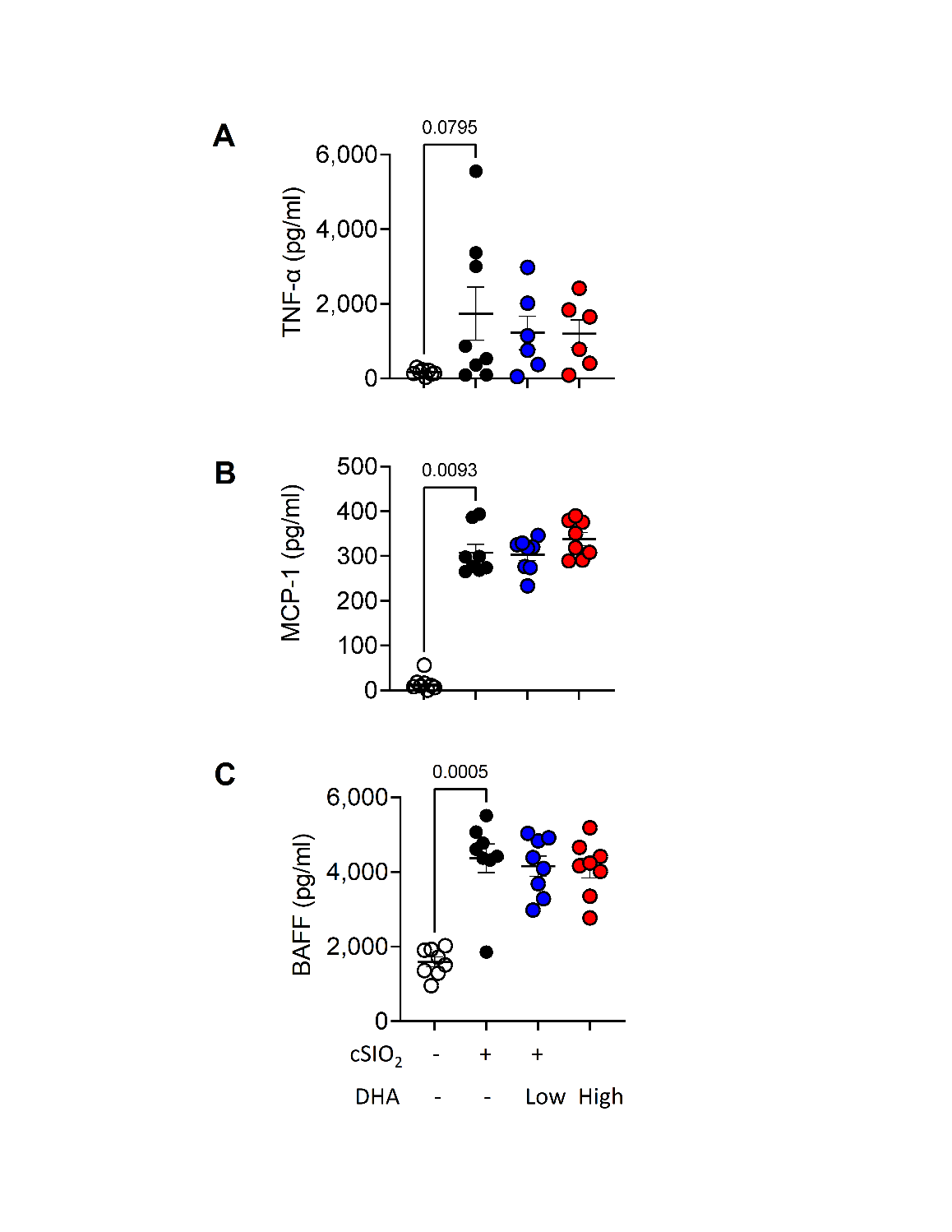


**Supplementary Figure 3.** **cSiO_2_-induced cytokine secretion in the BALF of NZBWF1 mice is not affected by low or high DHA diets.** Panels show secretion of **(A)** TNF-α, **(B)** MCP-1, and **(C)** BAFF expression. Data are presented as mean ± SEM (n=8).


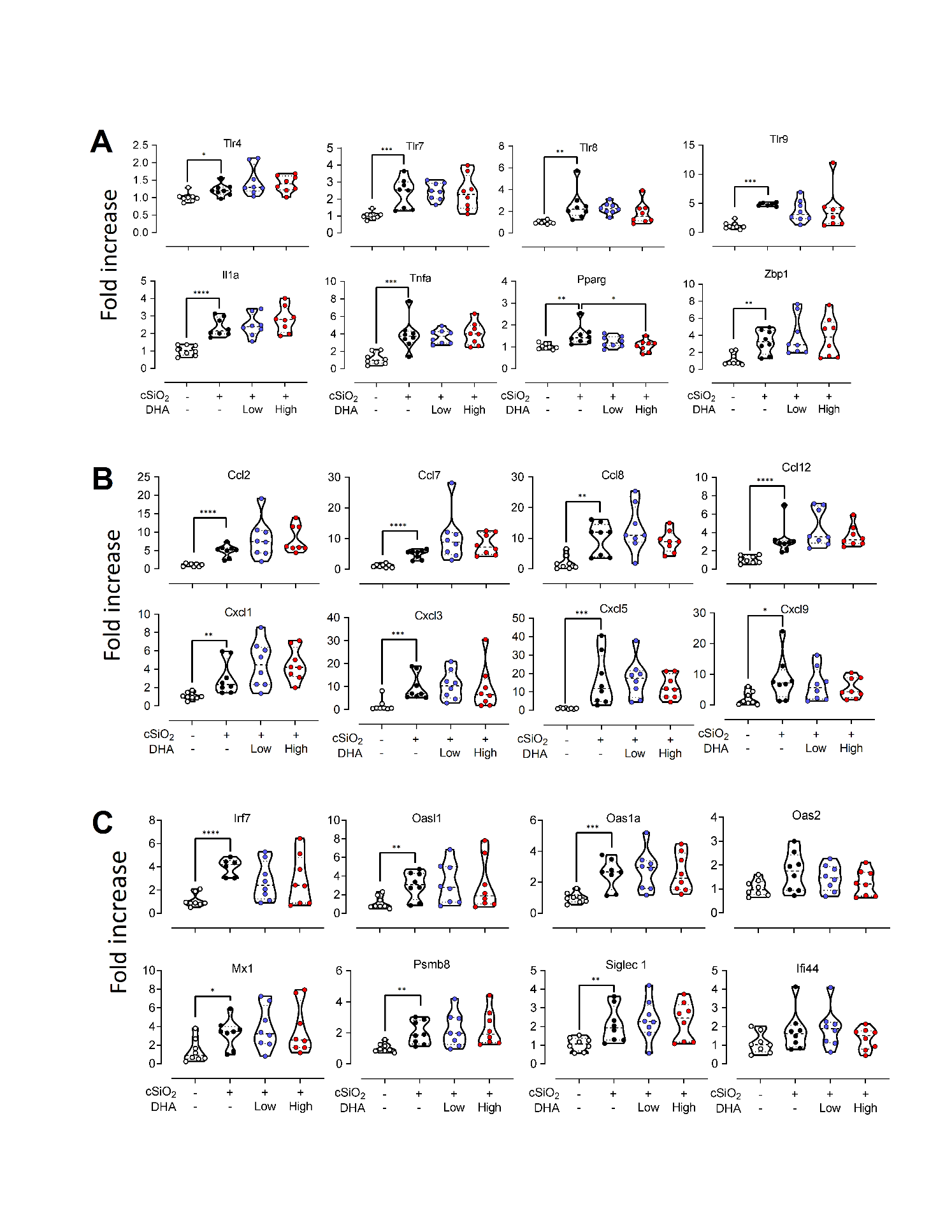


**Supplementary Figure 4****.** **cSiO_2_-induced gene expression in the lungs of NZBWF1 mice is not affected by low or high DHA diets.** Panels show fold change expression of **(A)** inflammatory, **(B)** chemokine, **(C)** interferon-related gene expression. Data are presented as mean ± SEM (n=8). *** p<.001, ** p<.01, *, p<0.05.


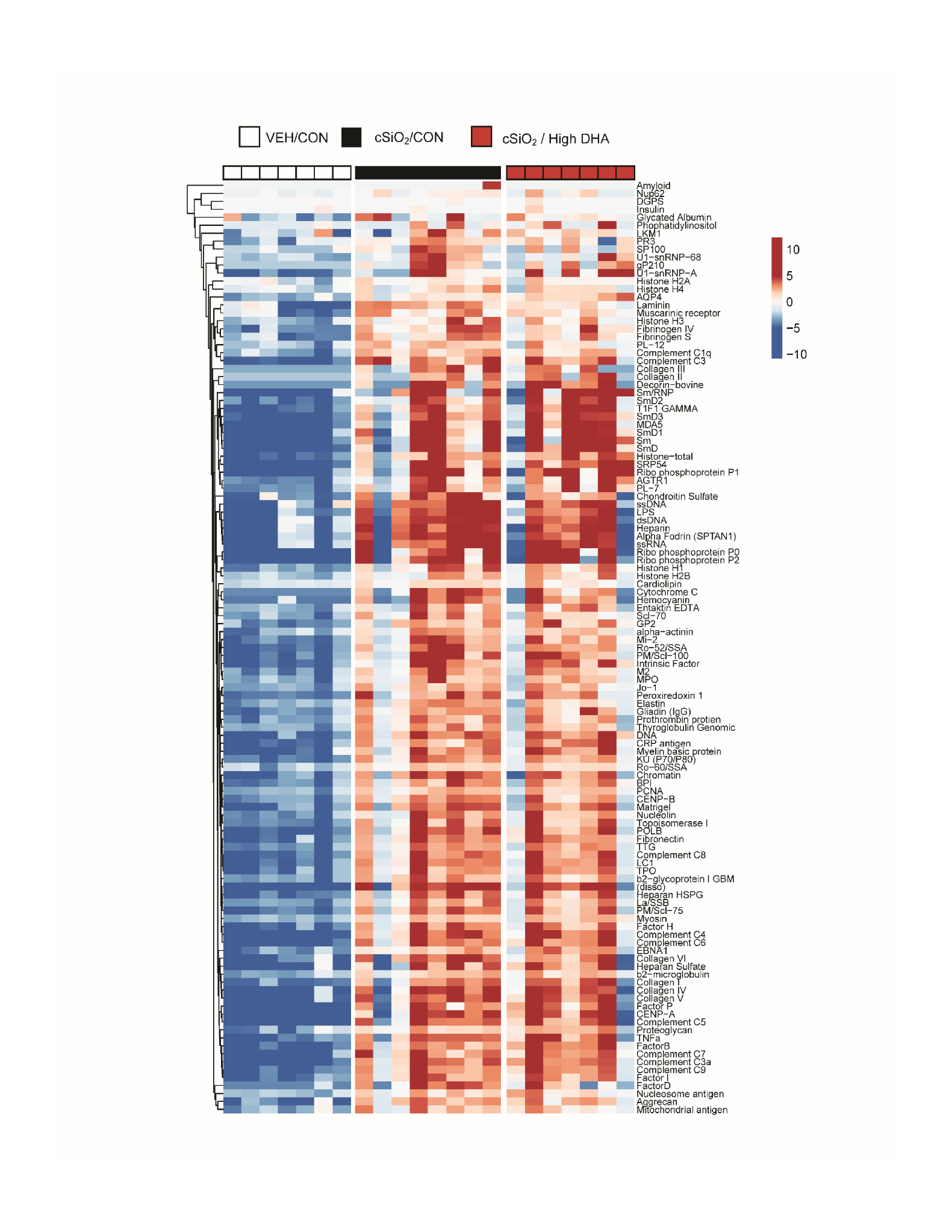


Supplemental Figure 3. Effects of DHA intake on cSiO_2_-induced IgG AAb responses in BALF. Heat maps with unsupervised clustering (Euclidian distance method) depict Ab-score values for IgA AAbs for 122 AAgs. White, black, and red in the top bar indicates the VEH/ CON, cSiO_2_/ CON, and cSiO_2_/ High DHA groups. Scale bar values reflect the range of variance-stabilized Ab scores, which were centered across rows.

**Supplementary Table 1.** Lung fatty acid content of experimental groups

|  |  | **Experimental Group** | | | | | | | | |
| --- | --- | --- | --- | --- | --- | --- | --- | --- | --- | --- |
|  |  | **cSiO_2_/CON** | | | **cSiO_2_/low DHA** | | | **cSiO_2_/high DHA** | | |
| **Common Name** | **Formula** | (% of total fatty acids) | | | | | | | | |
| Lauric | C12:0 | 0.02 | ± | 0.01 | 0.02 | ± | 0.01 | 0.03 | ± | 0.01 |
| Myristic | C14:0 | 1.62 | ± | 0.59 | 2.29 | ± | 0.47* | 2.52 | ± | 0.41** |
| Palmitic | C16:0 | 45.05 | ± | 8.46 | 51.32 | ± | 7.55 | 51.72 | ± | 4.94 |
| Palmitoleidic | C16:1ω7t | 0.07 | ± | 0.06 | 0.03 | ± | 0.00* | 0.03 | ± | 0.00* |
| Palmitoleic | C16:1ω7c | 4.27 | ± | 0.51 | 3.96 | ± | 0.31 | 4.34 | ± | 0.36 |
| Stearic | C18:0 | 5.11 | ± | 1.03 | 3.54 | ± | 0.56*** | 3.47 | ± | 0.54*** |
| Elaidic | C18:1ω9t | 0.00 | ± | 0.00 | 0.00 | ± | 0.00 | 0.00 | ± | 0.00 |
| Oleic | C18:1ω9c | 19.35 | ± | 4.25 | 16.92 | ± | 7.04 | 15.70 | ± | 4.21 |
| Vaccinic | C18:1ω7 | 1.51 | ± | 0.33 | 0.98 | ± | 0.40* | 0.93 | ± | 0.25* |
| Linoleic | C18:2ω6 | 4.19 | ± | 1.46 | 4.29 | ± | 0.98 | 3.83 | ± | 0.73 |
| Arachidic | C20:0 | 0.02 | ± | 0.01 | 0.02 | ± | 0.00 | 0.01 | ± | 0.00 |
| Gamma-linolenic | C18:3ω6 | 0.09 | ± | 0.03 | 0.05 | ± | 0.01* | 0.04 | ± | 0.01** |
| Linolenic | C18:3ω3 | 0.24 | ± | 0.08 | 0.14 | ± | 0.06* | 0.12 | ± | 0.04** |
| Eicosanoic | C20:1ω9 | 0.00 | ± | 0.00 | 0.00 | ± | 0.00 | 0.00 | ± | 0.00 |
| Conjugated Linoleic | CLA 9c,11t | 0.02 | ± | 0.01 | 0.01 | ± | 0.01 | 0.01 | ± | 0.01 |
| Eicosadienoic | C20:2ω6 | 0.09 | ± | 0.01 | 0.08 | ± | 0.01* | 0.06 | ± | 0.01*** |
| Eicosatrienoic | C20:3ω9 | 0.12 | ± | 0.06 | 0.04 | ± | 0.02** | 0.04 | ± | 0.01*** |
| Benehinic | C22:0 | 0.02 | ± | 0.00* | 0.01 | ± | 0.00* | 0.01 | ± | 0.00** |
| Dihomo-Gamma-linolenic | C20:3ω6 | 0.38 | ± | 0.16 | 0.31 | ± | 0.07 | 0.32 | ± | 0.07 |
| Arachidonic | C20:4ω6 | 8.00 | ± | 2.47 | 2.77 | ± | 0.27*** | 1.39 | ± | 0.17*** |
| Eicosapentaenoic | C20:5ω3 | 0.01 | ± | 0.01 | 0.32 | ± | 0.09*** | 0.86 | ± | 0.16*** |
| Lignoceric | C24:0 | 0.04 | ± | 0.01 | 0.03 | ± | 0.00 | 0.03 | ± | 0.00* |
| Nervonic | C24:1ω9 | 0.10 | ± | 0.02 | 0.06 | ± | 0.01* | 0.07 | ± | 0.01* |
| Adrenic | C22:4ω6 | 2.32 | ± | 1.13 | 0.25 | ± | 0.04** | 0.07 | ± | 0.01** |
| Docosapentaenoic ω6 | C22:5ω6 | 0.78 | ± | 0.29 | 0.01 | ± | 0.00* | 0.00 | ± | 0.00*** |
| Docosapentaenoic ω3 | C22:5ω3 | 0.13 | ± | 0.04 | 0.39 | ± | 0.05*** | 0.57 | ± | 0.12*** |
| Docosahexanoic | C22:6ω3 | 1.20 | ± | 0.36 | 5.57 | ± | 0.47*** | 7.34 | ± | 0.34*** |
| ∑ Unknown | | 0.98 | ± | 0.52 | 1.13 | ± | 0.12 | 1.30 | ± | 0.13 |
| ∑ SFA | | 51.89 | ± | 8.16 | 57.23 | ± | 7.41 | 57.79 | ± | 4.74 |
| ∑ MUFA | | 25.30 | ± | 4.29 | 21.95 | ± | 7.42* | 21.06 | ± | 4.34 |
| ∑ n-3 PUFA | | 1.57 | ± | 0.44 | 6.42 | ± | 0.45*** | 8.89 | ± | 0.47*** |
| ∑ n-6 PUFA | | 15.85 | ± | 5.32 | 7.75 | ± | 1.06** | 5.70 | ± | 0.73** |
| EPA+DHA | | 1.20 | ± | 0.36 | 5.89 | ± | 0.51*** | 8.19 | ± | 0.47*** |
| % ω3 in HUFA | | 10.32 | ± | 1.06 | 64.50 | ± | 2.15*** | 82.45 | ± | 1.30*** |

Determined by GLC. All fatty acids represented as percent of total fatty acids and as mean ± SEM, n=8. The data from animals in the Veh/Con group were not included due instrumental error during analysis. Asterisks indicate significantly different than cSiO_2_/CON: **p*<0.05, ***p*<0.01, ****p*<0.001.

**Supplementary Table 2.** Kidney fatty acid content of experimental groups

|  |  | **Experimental Group** | | | | | | | | | | | |
| --- | --- | --- | --- | --- | --- | --- | --- | --- | --- | --- | --- | --- | --- |
|  |  | **Veh/CON** | | | **cSiO_2_/CON** | | | **cSiO_2_/low DHA** | | | **cSiO_2_/high DHA** | | |
| **Common Name** | **Formula** | (% of total fatty acids) | | | | | | | | | | | |
| Lauric | C12:0 | 0.04 | ± | 0.00 | 0.03 | ± | 0.01 | 0.04 | ± | 0.02 | 0.06 | ± | 0.05 |
| Myristic | C14:0 | 0.76 | ± | 0.15 | 0.76 | ± | 0.09 | 0.57 | ± | 0.35 | 0.77 | ± | 0.53 |
| Palmitic | C16:0 | 20.21 | ± | 0.82 | 20.13 | ± | 1.99 | 20.71 | ± | 1.60 | 21.77 | ± | 1.31 |
| Palmitoleidic | C16:1ω7t | 0.04 | ± | 0.04 | 0.03 | ± | 0.01 | 0.02 | ± | 0.01 | 0.02 | ± | 0.01 |
| Palmitoleic | C16:1ω7c | 7.36 | ± | 2.43 | 7.02 | ± | 2.35 | 3.88 | ± | 3.07 | 3.71 | ± | 2.96 |
| Stearic | C18:0 | 5.97 | ± | 3.14 | 6.01 | ± | 2.51 | 9.37 | ± | 4.89 | 8.72 | ± | 4.79 |
| Elaidic | C18:1ω9t | 0.04 | ± | 0.01 | 0.05 | ± | 0.01 | 0.04 | ± | 0.01 | 0.04 | ± | 0.01 |
| Oleic | C18:1ω9c | 38.32 | ± | 9.50 | 38.96 | ± | 8.40 | 28.18 | ± | 15.42 | 28.14 | ± | 15.63 |
| Vaccinic | C18:1ω7 | 2.76 | ± | 0.17 | 2.64 | ± | 0.09 | 2.11 | ± | 0.17*** | 2.04 | ± | 0.28*** |
| Linoleic | C18:2ω6 | 8.72 | ± | 0.88 | 8.65 | ± | 1.21 | 10.16 | ± | 1.00* | 10.42 | ± | 1.41* |
| Arachidic | C20:0 | 0.04 | ± | 0.01 | 0.05 | ± | 0.01 | 0.07 | ± | 0.02* | 0.06 | ± | 0.02 |
| Gamma-linolenic | C18:3ω6 | 0.06 | ± | 0.02 | 0.06 | ± | 0.02 | 0.03 | ± | 0.01*** | 0.02 | ± | 0.01*** |
| Linolenic | C18:3ω3 | 0.08 | ± | 0.02 | 0.08 | ± | 0.03 | 0.07 | ± | 0.04 | 0.06 | ± | 0.03 |
| Eicosanoic | C20:1ω9 | 0.27 | ± | 0.02 | 0.29 | ± | 0.03 | 0.26 | ± | 0.05 | 0.23 | ± | 0.04* |
| Conjugated Linoleic | CLA 9c,11t | 0.03 | ± | 0.00 | 0.04 | ± | 0.01 | 0.03 | ± | 0.01 | 0.03 | ± | 0.01 |
| Eicosadienoic | C20:2ω6 | 0.09 | ± | 0.03 | 0.09 | ± | 0.02 | 0.13 | ± | 0.04 | 0.10 | ± | 0.04 |
| Eicosatrienoic | C20:3ω9 | 0.09 | ± | 0.01 | 0.10 | ± | 0.02 | 0.06 | ± | 0.03* | 0.05 | ± | 0.03** |
| Benehinic | C22:0 | 0.01 | ± | 0.00 | 0.01 | ± | 0.01 | 0.02 | ± | 0.01 | 0.01 | ± | 0.01 |
| Dihomo-Gamma-linolenic | C20:3ω6 | 0.30 | ± | 0.16 | 0.33 | ± | 0.14 | 0.70 | ± | 0.41 | 0.64 | ± | 0.38 |
| Arachidonic | C20:4ω6 | 9.61 | ± | 6.14 | 9.38 | ± | 4.83 | 10.19 | ± | 6.19 | 5.14 | ± | 3.51 |
| Eicosapentaenoic | C20:5ω3 | 0.01 | ± | 0.01 | 0.01 | ± | 0.01 | 1.05 | ± | 0.79* | 3.22 | ± | 2.15*** |
| Lignoceric | C24:0 | 0.04 | ± | 0.03 | 0.03 | ± | 0.01 | 0.05 | ± | 0.02 | 0.05 | ± | 0.03 |
| Nervonic | C24:1ω9 | 0.08 | ± | 0.09 | 0.03 | ± | 0.01^#^ | 0.05 | ± | 0.02 | 0.04 | ± | 0.02 |
| Adrenic | C22:4ω6 | 0.31 | ± | 0.20 | 0.38 | ± | 0.19 | 0.10 | ± | 0.04** | 0.02 | ± | 0.01** |
| Docosapentaenoic ω6 | C22:5ω6 | 0.54 | ± | 0.34 | 0.48 | ± | 0.29 | 0.01 | ± | 0.01* | 0.00 | ± | 0.00*** |
| Docosapentaenoic ω3 | C22:5ω3 | 0.08 | ± | 0.05 | 0.08 | ± | 0.04 | 0.28 | ± | 0.13** | 0.39 | ± | 0.21** |
| Docosahexanoic | C22:6ω3 | 2.58 | ± | 1.67 | 2.27 | ± | 1.27 | 9.23 | ± | 6.06* | 11.66 | ± | 7.41* |
| ∑ Unknown | | 0.34 | ± | 0.15 | 0.56 | ± | 0.27 | 1.00 | ± | 0.39 | 1.00 | ± | 0.56 |
| ∑ SFA | | 27.06 | ± | 3.21 | 27.02 | ± | 3.67 | 30.83 | ± | 4.96 | 31.44 | ± | 3.90 |
| ∑ MUFA | | 48.88 | ± | 11.65 | 49.02 | ± | 9.56 | 34.55 | ± | 17.81 | 34.23 | ± | 18.24 |
| ∑ n-3 PUFA | | 2.75 | ± | 1.71 | 2.45 | ± | 1.30 | 10.63 | ± | 6.90* | 15.33 | ± | 9.73* |
| ∑ n-6 PUFA | | 19.63 | ± | 6.73 | 19.37 | ± | 4.94 | 21.32 | ± | 5.89 | 16.36 | ± | 4.17 |
| EPA+DHA | | 2.59 | ± | 1.67 | 2.29 | ± | 1.28 | 10.28 | ± | 6.82 | 14.88 | ± | 9.55** |
| % ω3 in HUFA | | 19.44 | ± | 0.43 | 18.34 | ± | 1.84^##^ | 47.12 | ± | 4.99*** | 71.13 | ± | 4.22*** |

Determined by GLC. All fatty acids represented as percent of total fatty acids and as mean ± SEM, n=8. Number signs indicate significantly different than Veh/CON: ##, *p*<0.01. Asterisks indicate significantly different than cSiO_2_/CON: * *p*<0.05, ***p*<0.01, ****p*<0.001.
